# Nuclear fallout provides a new link between aPKC and polarized cell trafficking

**DOI:** 10.1186/s12915-016-0253-6

**Published:** 2016-04-18

**Authors:** Francisco J. Calero-Cuenca, José Manuel Espinosa-Vázquez, Miguel Reina-Campos, María T. Díaz-Meco, Jorge Moscat, Sol Sotillos

**Affiliations:** CABD, CSIC/JA/UPO, Campus Universidad Pablo de Olavide, Ctra. De Utrera Km. 1, Seville, 41013 Spain; Sanford-Burnham Medical Research Institute, La Jolla, CA 92037 USA

**Keywords:** aPKC, Cell polarity, Cell trafficking, Nuclear fallout

## Abstract

**Background:**

Cell polarity, essential for cell physiology and tissue coherence, emerges as a consequence of asymmetric localization of protein complexes and directional trafficking of cellular components. Although molecules required in both processes are well known their relationship is still poorly understood.

**Results:**

Here we show a molecular link between Nuclear Fallout (Nuf), an adaptor of Rab11-GTPase to the microtubule motor proteins during Recycling Endosome (RE) trafficking, and aPKC, a pivotal kinase in the regulation of cell polarity. We demonstrate that aPKC phosphorylates Nuf modifying its subcellular distribution. Accordingly, in *aPKC* mutants Nuf and Rab11 accumulate apically indicating altered RE delivery. We show that aPKC localization in the apico-lateral cortex is dynamic. When we block exocytosis, by means of exocyst-sec mutants, aPKC accumulates inside the cells. Moreover, apical aPKC concentration is reduced in *nuf* mutants, suggesting aPKC levels are maintained by recycling.

**Conclusions:**

We demonstrate that active aPKC interacts with Nuf, phosphorylating it and, as a result, modifying its subcellular distribution. We propose a regulatory loop by which Nuf promotes aPKC apical recycling until sufficient levels of active aPKC are reached. Thus, we provide a novel link between cell polarity regulation and traffic control in epithelia.

**Electronic supplementary material:**

The online version of this article (doi:10.1186/s12915-016-0253-6) contains supplementary material, which is available to authorized users.

## Background

Cells must be polarized to exert their functions. Establishment of cell polarity involves the trafficking machinery, which controls sorting of the molecules to their final destination, polarizing the organelles, the cortex and the extracellular membrane. The Par-6/Par-3/aPKC, the Crumbs/PATJ/PALS1 and the Scrib/Dlg/Lgl complexes control cell polarity and are conserved from invertebrates to vertebrates [1, 2]. Depending on the tissue, these complexes interact with different proteins. As a result, the mechanisms maintaining polarity vary from one tissue to another. These complexes also regulate cell signalling [3] and trafficking [2]. Thus, members of the polarity complexes modulate endocytosis [4] recycling and exocytosis [5–7]. However, how polarity complexes control trafficking routes and molecule delivery is mostly unknown.

We show that Nuclear Fallout (Nuf) and the atypical PKC (aPKC) are part of a new mechanism linking cell polarity with intracellular trafficking. Nuf belongs to the Rab11-family interacting proteins (FIPs), which bind and control the movement of the recycling endosome (RE) GTPase Rab11 through the microtubule cytoskeleton [8]. aPKC regulates cell polarity through the phosphorylation of various proteins modifying their behaviour or subcellular distribution. We demonstrate that aPKC phosphorylates Nuf affecting Nuf’s distribution and Rab11 and RE delivery. We show aPKC apico-lateral cortex levels are maintained by aPKC recycling in a Nuf-RE dependent manner. Thus, Nuf-aPKC act in a regulatory loop controlling Nuf-RE delivery and aPKC cortical levels.

## Results

### aPKC interacts and phosphorylates nuclear fallout

**Fig. 1:**
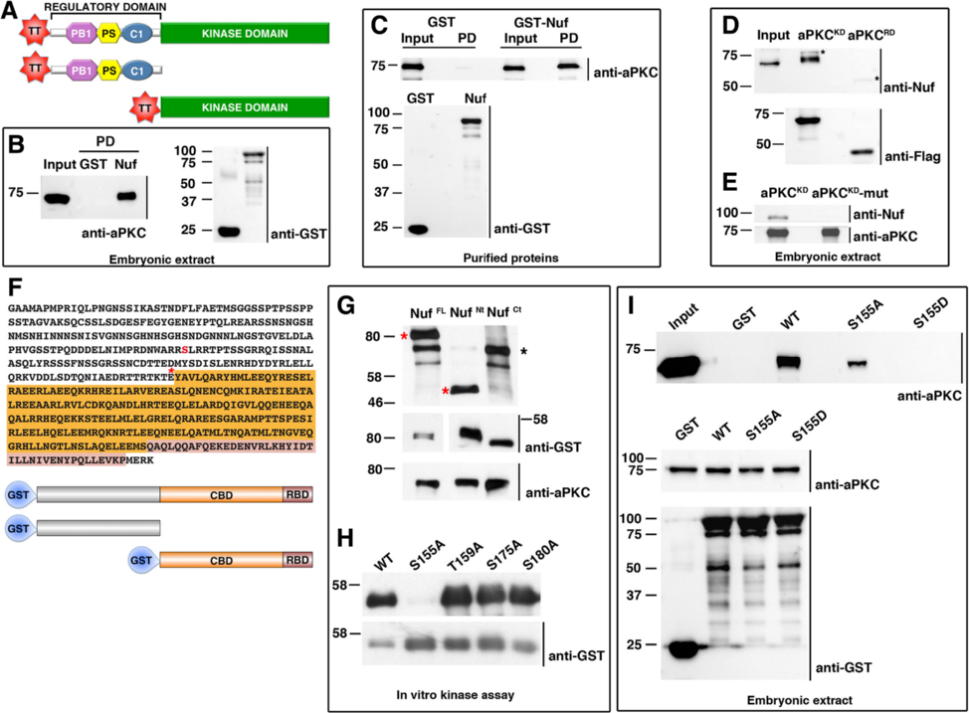
Nuf directly interacts with and is phosphorylated by aPKC. **a** aPKC full-length, regulatory and kinase domain Tetra-Tag (TT) constructs used in pull-down assays. **b** Pull-down (PD) assay of wild-type embryonic extracts with GST or GST-Nuf. Immunoblotting of bound proteins with anti-aPKC show aPKC presence only in the Nuf-containing sample. **c** In vitro assay of aPKC binding to Nuf. Recombinant aPKC was incubated in vitro with recombinant purified GST or GST-Nuf. Immunoblotting with anti-aPKC shows direct binding of aPKC to Nuf. **d** The kinase domain of aPKC interacts with Nuf. Embryonic extracts were incubated with aPKC kinase (Flag-aPKC^KD^) or regulatory domain (Flag-aPKC^RD^) bound to magnetic beads. Immunoblotting with anti-Nuf shows Nuf only binds to the kinase domain. The observed higher molecular weight in Nuf bound to the kinase domain of aPKC is consistent with Nuf being phosphorylated by aPKC. *Asterisks* point to aPKC versions used in the pull-downs cross-reacting with anti-Nuf. **e** Nuf only binds active aPKC. Wild type kinase domain (Flag-aPKC^KD^) and inactive kinase version (Flag-aPKC^KD-mut^) were purified with magnetic beads from embryonic extracts before incubation with purified recombinant Nuf-GST. Immunoblotting of bound proteins with anti-Nuf shows interaction with the wild type kinase domain. **f** Amino acid sequence and schematic representation of Nuf full-length and variants fused to GST. *Asterisk* marks Nuf N terminal fragment end. Orange: coiled-coil domain (CBD). Pink: Rab11 binding domain (RBD). Ser155 is highlighted in red. **g** Autoradiography of Nuf-GST variants subjected to aPKCz phosphorylation in vitro. Only full-length and N-terminal Nuf were phosphorylated (*red asterisks*). *Black asterisk* marks auto-phosphorylated aPKC. Preference towards substrate phosphorylation caused a reduction in autophosphorylation activity of aPKC. **h** Nuf phosphorylation sites. In vitro aPKC kinase was unable to phosphorylate S155A. **i** Recombinant GST-wild-type (WT), non-phosphorylatable (S155A) or phosphomimetic (S155D) Nuf pull-down assays from embryonic extracts. Immunoblotting probed with anti-aPKC shows Nuf S155D cannot interact with aPKC. “Input” contains 10 % (**b**, **d**, **i**) or 5 % (**c**) of the extracts. **b**-**e**, **g**-**i**. *Lower panels* are loading controls. Blots were probed with the indicated antibodies. *aPKC* atypical PKC*, Nuf* nuclear fallout, *GST* glutathione S-transferase

aPKC is a Ser/Thr kinase regulated by its interaction with other proteins [9]. In a Tandem Affinity Purification biochemical screen [8] to identify new aPKC partners/substrates we isolated Nuf (Fig. 1a and Additional file 1: Figure S1). GST pull-down assays using embryonic extracts and GST-Nuf recombinant protein confirm a strong interaction with endogenous aPKC (Fig. 1b). Pull-down assays of GST-Nuf and aPKC recombinant proteins indicate a direct interaction (Fig. 1c).

aPKC has an auto-regulatory pseudosubstrate domain (RD) and a kinase domain (KD) (Fig. 1a, [9]). The aPKC KD interacts with Nuf (Fig. 1d) only when active, as a kinase inactive version of aPKC [10] was unable to bind recombinant Nuf (Fig. 1e). This prompted us to investigated whether Nuf was phosphorylated by aPKC. Nuf belongs to the Arfophilin family of proteins, which are the targets of different kinases, IKK among them [11]. Nuf can be subdivided into an amino-terminal domain and a carboxy-terminal domain composed of a coiled-coil domain and the Rab11 binding domain (Fig. 1f). In vitro kinase assays showed the full-length Nuf and the amino-terminal domain (1–241) were phosphorylated by aPKC (Fig. 1g) correlating with the amino-terminal region of Nuf interacting with aPKC (Additional file 1: Figure S1). Interestingly, the human homolog Rab11-FIP3 is also phosphorylated by aPKC ζ and λ/ι, the two aPKC homologs in vertebrates (Additional file 1: Figure S1), indicating the conservation of the interaction and phosphorylation.

Nuf’s phosphorylation sites by aPKC were mapped in an in vitro kinase assay coupled to MS/MS detection of phosphopeptides. Phosphorylation sites at residues Ser155, Thr159, Ser175 and Ser180 were found (Additional file 1: Figure S1). We generated Nuf S155A, T159A, S175A or S180A single mutants and subjected them to aPKC in vitro phosphorylation. S155A abolished Nuf phosphorylation signal revealing S155 as the main phosphorylation target of aPKC in vitro (Fig. 1h).

Next we tested whether mutation of this serine residue modified Nuf interaction with aPKC. A phospho-mimetic mutation changing Ser155 to Aspartic (Nuf FL-S155D) was compared in pull-down assays with the full length and non-phosphorylatable versions of Nuf (Nuf FL-WT and Nuf FL-S155A, respectively). As with other aPKC substrates that lose affinity for the kinase once phosphorylated [12, 13], the phospho-mimetic version of Nuf did not bind to aPKC (Fig. 1i). These results identify Nuf as a novel aPKC substrate.

### aPKC controls Nuf’s sub-cellular localization

**Fig. 2:**
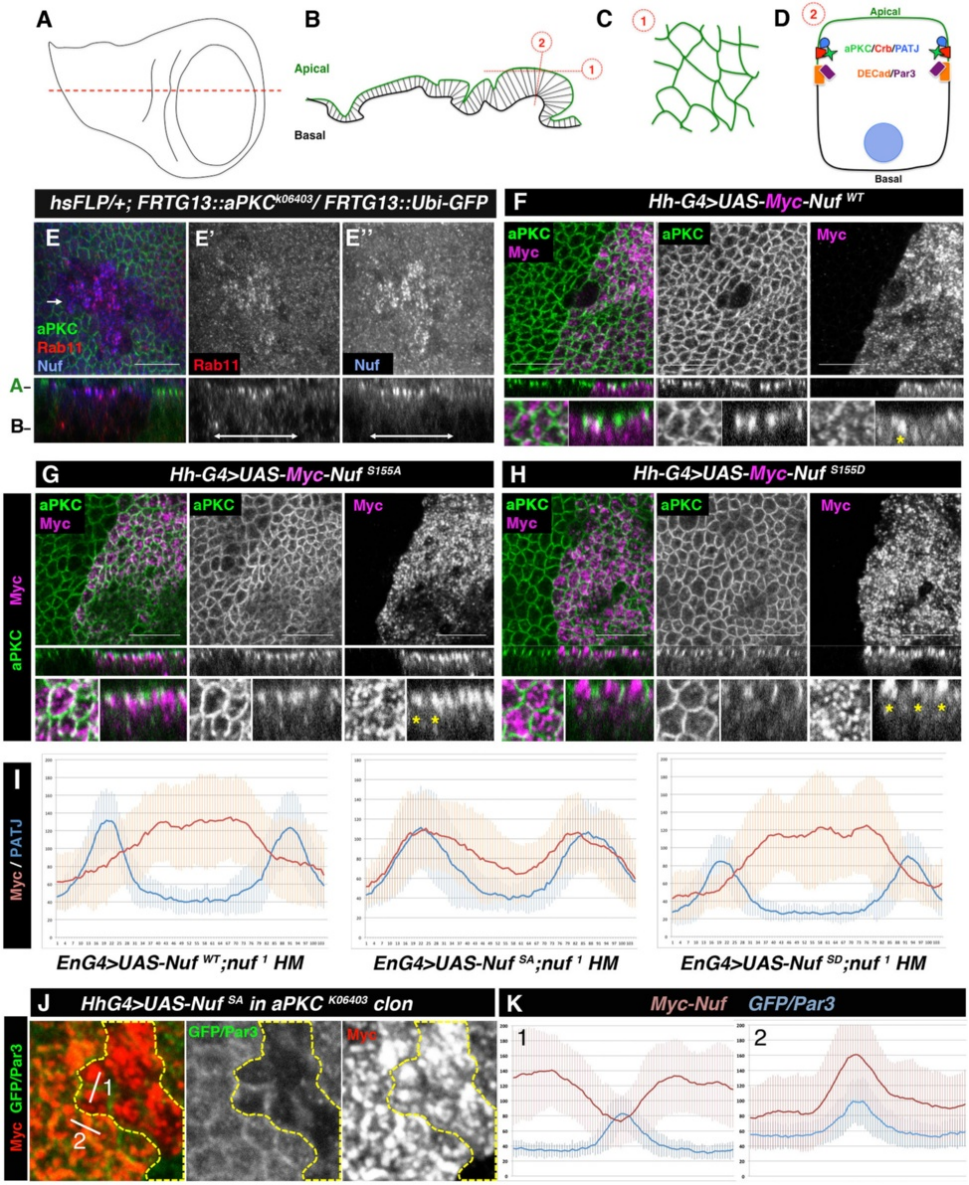
Nuf subcellular distribution is modified by aPKC phosphorylation. **a**-**b** Third instar imaginal wing disc (**a**) and a section is shown in (**b**). **c**-**d** Representation of an apical (1) and a transversal view (2) showing in d the position of apical-lateral markers. **e** aPKC clones in wing discs (marked by the absence of aPKC, green) accumulate Rab11 (e’ and red in e) and Nuf (e” and blue in e) apically. **f**-**h** Subcellular distribution of wild-type (**f**), non-phosphorylatable (**g**) and phosphomimetic (**h**) Nuf in wing discs. Nuf driven by *hh*-Gal4 accumulates apically. Nuf^S155A^ (**g**, magenta) reaches the apico-lateral membrane, marked by aPKC (green, co-localization in white). Nuf^S155D^ is excluded from the apico-lateral membrane (**h**). In (**f**-**h**), *upper panels* show apical views, *medial panels* show sagittal views and *lower panels* are close-ups of the above. *Yellow asterisks* mark the apical distribution of the respective Myc-Nuf versions. Scale bar 10 μm. **i** Quantification of myc (red) compared to DPATJ (blue) levels in epithelia of *nuf* ^*1*^ homozygous wing disc expressing Myc-Nuf^WT^, Myc-Nuf^S155A^ or Myc-Nuf^S155D^. Picks of DPATJ mark cell membranes. **j** Apico-cortical accumulation of Nuf^S155A^ (red) is lost in aPKC-depleted cells marked by the absence of GFP (green, delimited by the *yellow dotted line*). Par3 (green) was used as membrane marker. **k** Quantification of Myc (red) and GFP/Par3 (blue) at the boundary between two clone cells (1) or two wild-type cells (2). *Nuf* nuclear fallout, *aPKC* atypical PKC, *GFP* green fluorescent protein.

Nuf is an adaptor of Rab11 to the dynein and kinesin microtubule motor complexes affecting RE distribution [8, 14]. To find out if aPKC regulated Nuf and Rab11 subcellular distribution we studied the imaginal wing disc pseudo-stratified epithelium (Fig. 2a-d). Nuf is found in a uniform punctate distribution in the wild-type disc (Fig. 2e). However, in mutant *aPKC* clones Nuf accumulates in the subapical region (Fig. 2e”). This accumulation is accompanied by Rab11 accumulation (Fig. 2e’).

In embryonic epithelia, loss of aPKC is related with loss of apico-basal polarity. However, the accumulation of Nuf and Rab11 in the sub-apical region suggests aPKC mutant wing disc cells conserve certain polarity. We confirmed this by looking at other cell polarity markers. At the moment when we analysed Nuf localization in *aPKC* clones, the adherens junctions markers were still localized at the membrane as shown by E-Cadherin (Additional file 2: Figure S2) or Par-3 (Additional file 2: Figure S2). The sub-apical membrane determinant Crb is lost from the membrane of most of the cells in the clone, however some of them still retain Crb in the membrane (Additional file 2: Figure S2). It is noteworthy that in null mutant clones for other polarity determinants, such as Crb or Par-3, aPKC localization was not severely disrupted and Nuf was unaffected (Additional file 2: Figure S2). These results point to aPKC loss as the direct cause for Nuf’s subapical accumulation.

To examine whether the phosphorylation state of Nuf affected its subcellular localization we compared the distribution of the non-phosphorylatable and phospho-mimetic Nuf mutants. When over-expressed in wing discs, all Nuf proteins accumulated apically (Fig. 2f-h). However, whilst non-phosphorylatable Nuf accumulated close to the apico-lateral membrane, partly co-localizing with aPKC (Fig. 2g), phospho-mimetic Nuf was excluded from the lateral membrane (Fig. 2h). This localization is independent of the endogenous Nuf as the same distribution is detected in a *nuf* homozygous background (Fig. 2i).

Thus, the difference in Nuf distribution relies on its aPKC-phosphorylation state. In some polarity processes, phosphorylation induces binding to the cytoplasmic protein 14-3-3 [15] resulting in cytoplasmic protein retention [16–19]. This was not the case for Nuf, as although Nuf^WT^ and, to a lesser extent, Nuf^S155A^ and Nuf^S155D^ can bind 14-3-3 (Additional file 3: Figure S3) this binding was not modified by aPKC phosphorylation (Additional file 3: Figure S3).

We tested if the loss of affinity of phosphorylated Nuf for aPKC could be the cause for Nuf’s apical distribution by inducing *aPKC* null clones in cells expressing Myc-Nuf^S155A^. As shown in Fig. 2j-k, the cortical distribution of Nuf^S155A^ becomes cytosolic in *aPKC* mutant cells. This and the biochemical data (Fig. 1i) indicate that aPKC controls the apical distribution of Nuf by inhibiting via phosphorylation its apico-lateral accumulation.

### Non-phosphorylatable Nuf modifies aPKC apical levels

We examined whether aPKC affects Nuf localization by modifying its affinity for its known binding partners: Rab11 and the microtubule cytoskeleton motors dynein and kinesin [14, 20]. In pull-down assays we found Rab11 bound to wild type, non-phosphorylatable and phospho-mimetic Nuf with similar affinities. Similarly, Nuf phosphorylation did not affect its binding to kinesin and DLIC and dynactin, subunits of the dynein-dynactin complex. Moreover, these bindings were not competed by aPKC (Additional file 3: Figure S3).

**Fig. 3:**
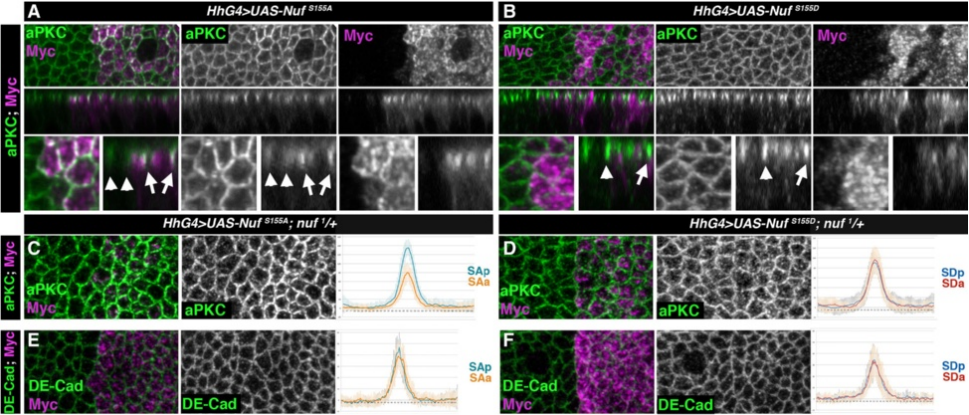
NufS155A increases aPKC apico-lateral membrane levels. **a**-**b** Wing discs of wild-type larvae expressing in the posterior compartment Myc-Nuf^S155A^ (**a**) or Myc-Nuf^S155D^ (**b**) stained for aPKC (green) or Nuf (anti-Myc, magenta). *Upper panels* apical views, *medial panels* sagittal views and *lower panels* close-up of the above. *Arrowheads* and *arrows* point to cortical aPKC in cells located in anterior or posterior compartment of disc. **c**-**f** Quantification of aPKC (**c**-**d**) and DE-Cad (**e**-**f**) levels in epithelia of *nuf* ^*1*^/+ heterozygous background wing disc expressing in the posterior Myc-Nuf^S155A^ (**c**, **e**) or Myc-Nuf^S155D^ (**d**, **f**). Fluorescence levels of aPKC or DE-Cad are shown comparing control anterior cells (orange and red) with posterior cells expressing Myc-Nuf^S155A^ or Myc-Nuf^S155D^ (blue). *Nuf* nuclear fallout, *aPKC* atypical PKC

To find if general delivery to the apico-lateral membrane was affected in Nuf mutants, we compared the distribution and levels of DE-Cad (a known RE cargo [21, 22]) and Par-3 markers of adherens junctions, and Crb and aPKC markers of the sub-apical region, in wing discs expressing UAS-Myc-Nuf^S155A^ or UAS-Myc-Nuf^S155D^. While neither of these proteins modified the levels or localization of DE-Cad, Par3 or Crb, expression of the non-phosphorylatable Nuf^S155A^ induced aPKC membrane accumulation (Fig. 3a). In contrast phosphomimetic Nuf^S155D^ caused no aPKC accumulation (Fig. 3b). To discard a possible interference of endogenous Nuf protein in the phenotypes, we repeated the experiment in *nuf* hetero- and homozygous mutants. In both cases the increase in aPKC levels in cells over-expressing Nuf^S155A^ was maintained (Fig. 3c-d and Additional file 4: Figure S4), but we did not observe any effect in the abundance or localization of the other markers (Fig. 3e-f and Additional file 4: Figure S4). These data indicate that the aPKC-dependent phosphorylation of Nuf exclusively prevents Nuf membrane localization without affecting its binding to the RE machinery and the RE delivery to the apico-lateral membrane and points to aPKC as a cargo of the RE.

### aPKC is recycled through the RE

The higher levels of aPKC observed in epithelia expressing Nuf-S155A suggest an active transport of aPKC via RE. In fact, mammalian aPKC transport in Rab11 positive RE has been described during the first steps of polarization of the mammary gland and in 3D MDCK cultures where it is necessary for cysts formation [5, 23]. However, active transport of aPKC has not been described in established epithelia. Moreover, a truncated form of *nuf* (*nuf*^*BRW*^ [24]) lacking the amino terminal region, therefore lacking the domain required for binding to and being phosphorylated by aPKC, overexpressed in a *nuf* mutant background accumulates apically colocalising with Rab11 (Additional file 5: Figure S5). In this condition aPKC can be detected in some of these Rab11 positive vesicles (Additional file 5: Figure S5). This data reinforces the transport of aPKC to the membrane via RE.

**Fig. 4:**
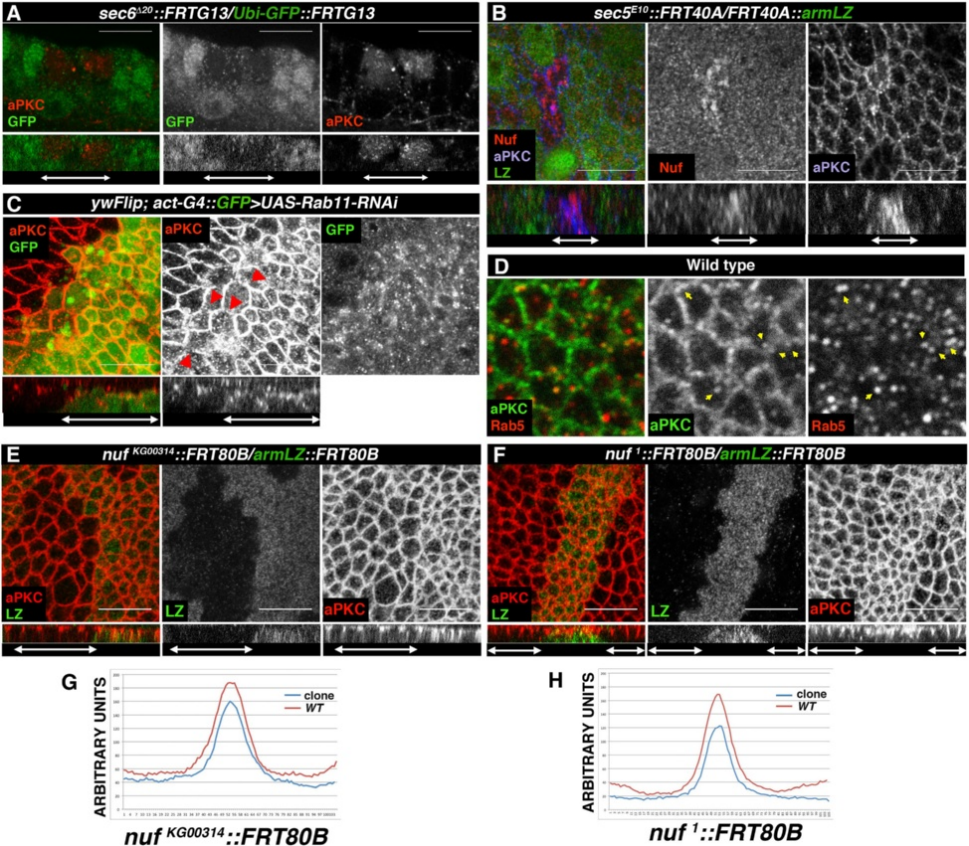
aPKC is recycled via Nuf-RE. **a**-**b** Confocal images of wing discs containing *sec6* (**a**) or *sec5* (**b**) clones. In the absence of sec6 (**a**) aPKC accumulates in the mutant cells (red, *right panel*). In *sec5* clones (**b**) aPKC (blue, *right panel*) and Nuf (red, *middle panel*) accumulate. **c** When Rab11 is silenced, aPKC (red) is disrupted from the apico-lateral cortex (*arrows*) accumulating in the cytoplasm (*right*). **d** Wing disc stained with aPKC (green) and the early recycling endosome marker Rab5 (red). *Arrows* point to co-stained endosomes. **e**-**f** aPKC (red) is reduced in *nuf* ^*KG00314*^ (**e**) or *nuf* ^*1*^ (f) mutant clones. **g**-**h** Fluorescence levels of aPKC comparing wild-type (red) with clone cells (blue) for *nuf* ^*KG00314*^ (**g**, *left*) or *nuf* ^*1*^ (**h**, right). Clones are marked by the absence of GFP (**a** and **b**) or β-gal (**e**-**f**) or expression of GFP (**c**) in green. (**a**-**c**, **e**-**f**) *Lower panels* show sagittal views of the clones and are marked by *arrows. aPKC* atypical PKC, *RE* recycling endosome, *GFP* green fluorescent protein

If aPKC transport to the apical membrane depends on RE, blocking exocytosis in mutants for *sec6* or *sec5* should increase cytosolic aPKC. Although *sec6* mutant cells have very low viability, the few clones recovered accumulated aPKC and Nuf (Fig. 4a, red) and other recycling cargoes, such as Delta [25] and not shown). We obtained similar results with *sec5* (Fig. 4b, blue). Accordingly, when eliminating Rab11 with a specific RNAi we observed aPKC was lost from the cell junctions and accumulated sub-apically inside the cell (Fig. 4c). Moreover, we found vesicles co-stained with aPKC and Rab5, an endosomes marker, supporting an active endocytosis of aPKC and reinforcing the idea of aPKC recycling (Fig. 4d).

Considering that blocking exocytosis, interfering with sec6 or sec5, induces accumulation of aPKC, Rab11 and Nuf (Fig. 4 and [26]), that blocking the traffic of the RE by elimination of Rab11 generates aPKC accumulation in the cell and that aPKC accumulates in the apico-lateral cortex when a non-phosphorylatable version of Nuf is overexpressed, our data suggest an active movement of aPKC to the membrane through Nuf-positive RE. To confirm this hypothesis, we induced *nuf*^*1*^ or *nuf*^*KG00314*^mutant clones and observed a reduction of aPKC levels at the apical cortex (Fig. 4e-h), whilst other apical determinants as DPATJ or the adherens junctions markers Par3 and DE-Cad are unaffected (Additional file 6: Figure S6). Together, our data strongly indicate that aPKC is recycled to the membrane via Nuf-Rab11-RE.

## Discussion

Recent research has shown the relationship between apico-basal cell polarity and polarized cellular trafficking. Here, we uncovered a novel direct link between apico-basal polarity and trafficking at the level of the RE, mediated by aPKC and Nuf. Nuf is phosphorylated by aPKC and this phosphorylation controls the trafficking of the RE to the apico-lateral domain. We also show an aPKC recycling mechanism dependent on Nuf-Rab11-RE exists in the mature epithelia of the imaginal wing disc. Nuf is a hyperphosphorylated protein. Recently Otani et al. demonstrated that the phosphorylation state of Nuf by IKKε regulates the subcellular distribution of Nuf and RE in the chaeta [11]. Here we unravel a new mechanism to control Nuf localization by aPKC-dependent phosphorylation.

aPKC function is mediated by phosphorylation of different substrates. In apico-basal polarity, aPKC refines the distribution of target proteins as Par-3/Baz or Crb [10, 18, 27] or locally excludes target proteins as Par-1, Pins, Lgl, Yurt or Numb [17, 19, 28–33]. Nuf distribution is also determined by aPKC-dependent phosphorylation. When aPKC is absent Nuf accumulates apically. Moreover, while the non-phosphorylatable Nuf accumulates adjacent to the lateral membrane the phosphomimetic version of Nuf is displaced from the apico-lateral cortex. This distribution is due to the binding affinity of Nuf to aPKC, as phosphomimetic Nuf^S155D^ in pull-down assays cannot bind aPKC and avoids the apico-lateral cortex when expressed in imaginal discs whilst the non-phosphorylatable version Nuf^S155A^ conserves the capacity to bind aPKC, accumulates in the apical cortex when expressed in epithelia and loses this distribution in the absence of aPKC. In fact, endogenous Nuf accumulates apically in aPKC mutant cells but avoids the apico-lateral cortex (Additional file 2: Figure S2) supporting the requirement of aPKC for apico-lateral distribution. However, in the wild type situation this interaction must be very transient as no membrane co-localization of aPKC and Nuf in the membrane can be detected. It has been described that aPKC phosphorylation can drive the interaction and retention in the cytoplasm of aPKC substrates by the protein 14-3-3/par5 [17, 19, 34, 35]. This is not the case with Nuf since, although Nuf binds 14-3-3 in vitro, aPKC dependent phosphorylation does not modify the binding. However, considering Nuf is a hyper-phosphorylated protein, 14-3-3 could modulate its function in other processes where Nuf could be involved. Interestingly, a function of 14-3-3 regulating Rab11 recycling endosomes has been described in *Caenorhabditiselegans* [36] and in *Drosophila* [37].

Considering that the main function of Nuf is to control the traffic of Rab11-associated recycling endosomes and aPKC phosphorylation or binding does not modify Nuf’s affinity for Rab11, dynein or kinesin complexes, by controlling Nuf’s subcellular distribution aPKC may affect the trafficking of Nuf-RE directed vesicles. Over-expression of different phosphorylated states of Nuf did not affect the distribution of DE-Cad, pointing to a DE-Cad-Rab11 recycling independent of Nuf in accordance with observations in the tracheal system where DE-Cad recycling depends on Rip11, the other arfophilin in *Drosophila* [22]. Par-3 distribution remains unaltered when NufS155A is overexpressed, suggesting that recycling of Par-3 is also Nuf independent. Our experiments did not detect any influence of Nuf on Crb localization. However, Crb membrane recycling has been shown to depend on the retromer and only in the newly synthetized membranes depends on Rab11 [2].

**Fig. 5:**
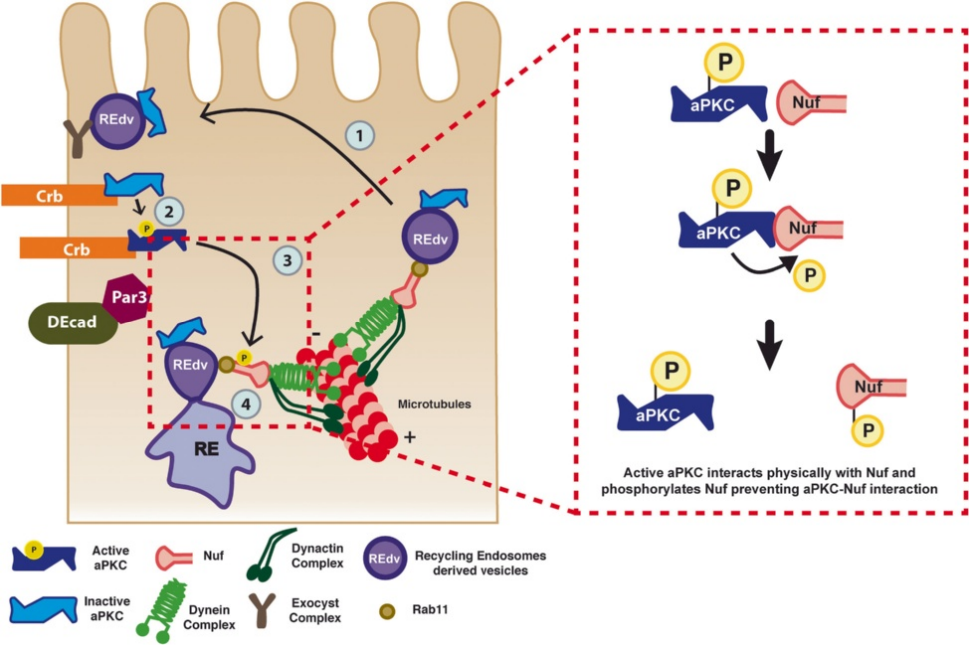
aPKC recycling in the epithelial cells. Schematic model of aPKC transport to the membrane in Rab11-Nuf RE vesicles (*left*): (1) Vesicles derived from the RE (REdv) containing aPKC move to the apico-lateral site via Rab11-Nuf. (2) aPKC interacts with the apical complexes and becomes active. (3) Active aPKC is able to interact with and phosphorylate Nuf. This phosphorylation release aPKC-Nuf interaction (*red dash-line box* on the *right*) (4) inhibiting its apico-lateral localization. *aPKC* atypical PKC, *RE* recycling endosome, *Nuf* nuclear fallout

In contrast, the levels of aPKC depend on aPKC-Nuf interaction. Expression of Nuf^WT^ or Nuf^S115D^ do not alter the levels of aPKC, but expression of non-phosphorylatable Nuf^S155A^ increases aPKC levels in the apico-lateral cortex. This suggests an active transport of aPKC to the apical membrane in Nuf-Rab11 RE. We obtained additional evidence in this direction by interfering with the general RE pathway overexpressing a truncated form of Nuf (Nuf^BRW^). We observed a significant increase in the number of Rab11 positive vesicles and the induction of strong phenotypes similar to Rab11-RNAi expression and suggesting a blockage of the recycling pathway. Under this condition, we observed a partial accumulation of aPKC in apical vesicles that are also Rab11 positive, further supporting a Nuf-dependent aPKC recycling mechanism (Additional file 5: Figure S5). However, unlike Nuf^S155A^, Nuf^BRW^ does not accumulate in the membrane, neither aPKC membrane levels are modified, indicating a failure at some level in the fusion of the vesicles, maybe due to the inability of Nuf^BRW^ to interact with the active aPKC in the membrane. The role of Nuf in the recycling of aPKC is consistent with the cytoplasmic accumulation of aPKC when we block exocytosis in exocyst mutants or RE-vesicle delivery by eliminating Rab11 (Fig. 4), and with the presence of vesicles co-stained with the endocytic endosome GTPase Rab5 and aPKC. Nuf mutant cells show lower levels of apical aPKC suggesting a Nuf-Rab11-RE-mediated transport of aPKC to the apical membrane and aPKC inhibits its own recycling by phosphorylation of Nuf displacing Nuf and Nuf associated-RE from the apico-lateral membrane (Fig. 5).

Thus, although other scenarios are possible (i.e. a passive transport of aPKC due to interaction with other apical determinant actively transported via Rab11-RE or forming complex of the RE as a recycling regulator), all our data strongly point to an apical recycling of aPKC via Nuf-Rab11 in *Drosophila*. Moreover, we suggest that aPKC inhibits its own recycling by phosphorylation of Nuf, displacing Nuf and Nuf-associated RE from the apico-lateral membrane (Fig. 5).

Among the most common apical sorting signal is the presence of N-glycans attached to the protein. Interestingly, *Drosophila* aPKC has two N-glycosylation sites (position 18–21 and 226–229 respectively) as in vertebrates (aPKC λ position 18–21 and 241–244) that could be responsible for apical delivery. Also, our data suggest aPKC is a regulator of ARE-Rab11 delivery by controlling Nuf apical cortex interaction. It is not clear how Rab11 docks to and fuses with the membrane. aPKC could help via Nuf to Rab11 membrane docking. Recently, the SNARE interactor protein Munc13-4 has been involved in Rab11 docking to the membrane [38]. It would be interesting to investigate if the *Drosophila* homolog unc 13-4A also controls Rab11 docking and if a relationship with aPKC exists.

This aPKC-dependent mechanism for membrane recycling may be evolutionarily conserved since FIP3, the human ortholog of Nuf, interacts with, and is phosphorylated by, aPKCλ (Additional file 1: Figure S1). Also, the aPKC Rab11-FIP3-dependent recycling in epithelial cells may be preserved in mature epithelia and not only adscript to lumen formation.

## Conclusions

Cells must be organized in different functional domains to maintain cell homeostasis and exert their functions. This is achieved through conserved proteins that polarize the cell or regulate directional trafficking of cellular vesicles. Both mechanisms must be coordinated but there is little information about their relationship. Here, we describe a new connexion between Nuf, an adaptor of Rab11-GTPase to the microtubule motor proteins in the recycling endosomes (RE) process, and aPKC, one of the main regulators of cell polarity. We demonstrate that aPKC phosphorylates Nuf, modifying Nuf subcellular distribution and, thus, RE delivery. We also show that aPKC’s apical recycling is maintained through Nuf-Rab11-RE. Thus, our results provide a novel link between cell polarity regulation and cellular traffic control.

## Methods

### Fly strains

We used the following stocks: Oregon R (wild-type), *nuf*^*1*^ [39], *nuf*^*KG00314*^ (flybase), *nuf*^*BRW*^ [24], *FRTG13::aPKC*^*K06403*^, *baz*^*4*^*::FRT19a*, *FRT82B::crb*^*1*^, *sec6*^*∆20*^*::FRTG13,/+* [40], *sec5*^*E15*^*::FRT40A* [26]. UAS-Rab11-RNAi (v22198) from the Vienna *Drosophila* RNAi Centre.

We used 69B*-Gal4*, *24B-Gal4*, *en-Gal4* and *Hh-Gal4* as driver lines.

### Immunohistochemistry

Imaginal discs were fixed for 20 minutes in paraformaldehyde 20 % followed by a second fixation of 20 minutes in paraformaldehyde 20 %–0.1 % TritonX-100.

The following primary antibodies were used: anti-Nuf (1:500, generated in this work as described in [11]), mouse and rabbit anti-aPKC (1:100 and 1:500, respectively) and rabbit anti-Myc (1:250) from Santa Cruz Biotechnology; mouse anti-Myc (Cell Signalling 1:500), rabbit anti-GFP (Molecular Probes, 1:300); anti-Dlg (1:100), anti-Crb (1:100) and anti-DECad (1:20) from Developmental Studies Hybridoma Bank, mouse anti-Myc (Cell Signalling, 1:500); anti-Rab11 (BD Biosciences, 1:100); anti-ßgal (Promega, 1:10.000), anti-Baz (1:500, this work), anti-DHC (1:500, [41]), anti-Rab5 (1:25, a gift from M. González-Gaitán) and anti-DPATJ (1:500, a gift from H. Bellen). Secondary antibodies were coupled to Alexa488, Alexa555 or Alexa647 (Molecular Probes).

Affinity-purified guinea pig antibody for Nuf was generated in this study by conventional methods. Briefly, *E. coli* BL21(DES)pLyS was transformed with pGEX-6P-1 Nuf FL and the protein overexpression was performed at 20 °C with 0.1 mM IPTG for 20 hours. The recovered cell pellets were lysed by sonication in PBS, 1 mM DTT and 1X complete protease inhibitors cocktail (Roche) buffer. After sonication, the lysate was incubated on ice with 1 % Triton-X100 for 30 minutes. The lysate was centrifuged (14,000 rpm, 4 °C, 30 minutes) and incubated with glutathione-Sepharose beads (Sigma) for 4 hours at 4 °C, the beads were washed three times with PBS and 1 mM DTT and twice with PBS, 1 mM DTT and 1 M NaCl. The protein bound to the beads was eluted with elution buffer (50mM Tris HCl pH 8.5, 0.1 %, Triton X-100, 100 mM NaCl, 1 mM DTT, 20 mM glutathione). The eluted material was lyophilised and sent to Genosphere Biotech to generate the antibody.

Affinity-purified rabbit antibody for Baz/Par-3 was generated in this study by transformation of BL21(DES)pLyS with pGEX-6P-1-Baz N1 [42] and following the same protocol as above with anti-Nuf.

Images were taken on a SP2-AOBS or a SPE Leica confocal microscopes and processed using FIJI and Adobe Photoshop programs.

### Quantification of antibody staining and statistics

A total of fifteen confocal images of 0.16 μm thickness of a wing imaginal disc were projected using the average intensity algorithm (ImageJ) and five cells were randomly selected in the anterior and posterior compartments. The fluorescence intensity (pixel grey intensity shown in arbitrary units) was measured along a line 3 μm long. Different samples (n = 5) were measured for each genotype (n = 25).

In Additional file 4: Figure S4d the differences between fluorescence at the membrane and the cytoplasm were calculated for each cell analysed (n = 25) in anterior or posterior and in the different backgrounds as indicated. Data are displayed as a box plot and the statistical significance was calculated using the non-parametric Wilcoxon test.

To analyse Nuf distribution (Fig. 2i) 15 confocal images of 0.16 μm thickness of a wing imaginal disc expressing the different Nuf transgenes were projected using the average intensity algorithm (ImageJ). A total of five cells were randomly selected and five different samples were measured for each genotype (n = 25 each). The myc fluorescence intensity (pixel grey intensity shown in arbitrary units) was measured along a line 3 μm long that encompassed the two opposite membrane cells. Myc fluorescence was compared to PATJ, an apico-lateral protein of the Crb complex.

Figure 2k shows the fluorescence levels of myc-NufSA, measured as described above, in the border between two aPKC mutant cells (1) or two sibling wild type cells (2). The border cell was defined and measured by Par-3 levels. Keep in mind that Par-3 and GFP (marker of wild type cells) are stained in green indicating the higher basal levels of Par3 in wild type cells.

### Pull-down assays and Western blots

To perform pull down experiments Nuf-GST fusion proteins were obtained from subclones in pGEX-6P-1 (Nuf FL) or pGEX2T (Nuf-NH and Nuf-CO). Purification was done as described in [11]. A total of 300 μg of protein extract from *Drosophila* embryos in lysis buffer (50 mM Tris HCl pH 7.5, 150 mM NaCl, 1 mM EGTA, 2 mM EDTA, 1 % Triton X-100, 5 mM DTT, 1 mM PMSF, 1 mM sodium orthovanadate, 1X complete protease inhibitor cocktail (Roche) and 10 mM β-glycerol phosphate) were incubated for 4 hours at 4 °C with 30 μg of glutathione-S-transferase (GST) or of the different GST-fusion proteins bound to glutathione beads as described in [10]. Complexes were washed three times using 50 mM Tris HCl pH 7.5, 50 mM NaCl and 0.1 % Triton X-100 and once with 50 mM Tris HCl pH 6.8. In vitro pulldown was performed incubating 20 μg of GST or Nuf-GST proteins bound to glutathione beads with 0.25 μg of PKCz (*PRKCz Recombinant Human Protein,* Life Technologies) in lysis buffer for 2 hours at 4 °C. Complexes were washed three times using PBS 1X supplemented with 0.5 M NaCl and once with 50 mM Tris HCl pH 6.8. The GST-tag of proteins expressed from pGEX-6P-1 plasmids was removed by PreScission protease (GE Healthcare) following the manufacturer’s protocol and used in vitro for the Rab11-GST pull-down assay as described in [10].

Expression and purification of the recombinant proteins GST-14-3-3ε and GST-DLIC was performed as described above for Nuf from pGEX-6P-1-14-3-3 and pGEX-6P-1-DLIC respectively.

#### Competitive pull-down assays

Kinesin and dynactin: 20 μg of GST, GST-NufNH wt, GST-NufNH S155A and GST-NufNH S155D fusion proteins were incubated with 300 μg of OR extract for 3 hours at 4 °C in lysis buffer (50 mM Tris HCl pH 7.5, 150 mM NaCl, 1 mM EGTA, 2 mM EDTA, 1 % Triton X-100, 5 mM DTT, 1 mM PMSF, 1 mM sodium orthovanadate, 1X complete protease inhibitor cocktail (Roche) and 10 mM β-glycerolphosphate). Beads were washed three times with 50 mM Tris HCl pH 7.5, 50 mM NaCl and 0.1 % Triton X-100 and incubated with 0, 50 or 200 μg of PKCz (PRKCz recombinant human protein, Life Technologies) for 1 hour at 4 °C. Beads were washed three times with IP15OO buffer and once with Tris HCl 50 mM pH 6.8.

GST-Rab11 and GST-DLIC: 20 μg of GST and GST-fusion proteins were incubated with 200 ng of NufFL wt, NufFL S155A and NufFL S155D proteins (GST was removed with Prescission protease, GE Healthcare) for 2 hours at 4 °C in Rab11 interaction buffer (PBS, 2 mM DTT, 1 mM sodium orthovanadate, 1 mM PMSF, 1X complete protease inhibitor cocktail) and DLIC interaction buffer (50 mM Tris HCl pH 7.5, 0.5 % Triton X-100, 150 mM NaCl, 10 % glycerol and 1 mM EDTA) respectively. Beads were washed three times with Rab11 interaction buffer supplemented with 50 mM NaCl for Rab11 assay and DLIC interaction buffer for DLIC assay. After that, beads were incubated with 0, 50 or 200 ng of PKCz in Rab11 interaction buffer or DLIC interaction buffer for 2 hours at 4 °C. Beads were washed three times with Rab11 interaction buffer supplemented with 50 mM NaCl or DLIC interaction buffer. In both cases, beads were finally washed with Tris HCl 50 mM pH 6.8.

Western blotting was done according to standard procedures using guinea pig anti-Nuf (1:5000), rabbit anti-aPKC (Santa Cruz Biotechnologies, 1:5000), anti-GST (Santa Cruz Biotechnologies, 1:3000), mouse anti-Flag (Sigma, 1:10000), rabbit anti-Kinesin (Cytoskeleton, 1:3000), anti-Glued (Dynactin, 1:1000 [43]), rabbit anti 14-3-3 (Santa Cruz Biotechnologies, 1:500) and mouse anti-α-Tubulin (Sigma, 1:10000).

### aPKC phosphorylation assay

This method has been adapted from [44]. For each reaction, 150 ng of recombinant substrate (GST-fusion proteins) was incubated with 140 ng of human recombinant PKCiota or PKCz produced in Sf9 cells (Moscat laboratory) in phosphorylation buffer {(35 mM Tris HCl pH 7.5, 10 mM MgCl_2_, 0.1 mM CaCl_2_, 0.5 mM EGTA, 1 mM DTT and 0.1 mM ATPγS (*Biolog*)}) for 1 hour at 30 °C. The reaction was stopped by adding 20 mM EDTA and samples were incubated with 2.5 mM PNMB (Abcam) for 1 hour at room temperature. The samples were analysed by SDS-PAGE and western blotting using anti-thiophosphate ester antibody (Abcam, 1:5000). For MS/MS detection of Nuf phosphopeptides, an in-vitro kinase assay was performed using 10 μg of GST-Nuf FL and 10 μg of PKCiota in 400uM ATP and 1x phosphorylation buffer. Phosphorylation products were digested and processed using LS-MS/MS analysis by the SBMRI Proteomic Service.

### Phosphorylated Nuf −14-3-3ε pull-down assay

A total of 20 μg of GST and GST-NufFL wt were incubated for 1 hour at room temperature with phosphorylation buffer adding ATP and 500 μg of PKCz only to the Nuf phosphorylated experiment. Beads were washed three times with 14-3-3 interaction buffer (25 mM Hepes pH 7.5, 12.5 mM MgCl_2_, 20 % glycerol, 0.1 % NP40, 150 mM KCl, 1 mM DTT) and were incubated with 500 ng of eluted 14-3-3ε (removing GST with Prescission protease) in 14-3-3 interaction buffer adding 30 μg of BSA for 3 hours at 4 °C. After that, beads were washed three times with 20 mM Tris HCl pH 8, 100 mM NaCl, 1 mM EDTA and 0.5 % NP40, and once with Tris HCl 50 mM pH 6.8.

Additional Methods, including primers used, are given in Additional file 7.

## Additional files

Additional file 1: Figure S1. Tandem Affinity Purification of aPKC. aPKC-Nuf interaction and phosphorylation. a. EZ-Blue staining of a gel loaded with the elution fractions obtained after Tandem Affinity Purification of aPKC from control embryonic extract (69B-Gal4) or embryos overexpressing TTaPKC FL (*69B-Gal4* driver). Nuf and known aPKC interacting proteins (Par-6, Par-1 and Lgl) were identified. Peptides corresponding to Nuf identified by mass spectrometry analysis are shown on the right. b. Pull-down assays from *Drosophila* embryonic protein extracts with GST, GST-Nuf FL or GST fused to the amino (Nt) or carboxy (Ct) terminal domains of Nuf. Immunoblotting of bound proteins with anti-aPKC shows the interaction of Nuf full-length and the amino terminal but not the carboxy terminal region of Nuf with aPKC. The *first lane* (Input) contains 10 % of the extract used in the assay. In the *middle panel*, extract was probed with anti-α-aPKC as a loading control. *Lower panel*: 10 % of the beads used in each pull-down assay were run in a gel and probed with anti-GST. c. To gain more insight into the phosphopeptide-to-protein mapping, we used two widely used software programs for peptide identification, namely MSGF+ and MaxQuant. While the former provides better protein sequence coverage, MaxQuant is more statistically robust. Note that phosphorylation of S155 on NUF was detected on both phosphopeptide-enriched and non-enriched samples while the other four phosphorylation sites of Nuf (S175, S181, S159, T163) were detected only after enrichment. Given the fact that LC-MS/MS system provides low attomoles sensitivity, it is likely that the stoichiometry of these phosphopeptides is extremely low. d. Rab11-FIP3, human orthologous of Nuf, is phosphorylated by human aPKCs, aPKC ζ and λ/ι, *red asterisks. Blue asterisk* marks aPKC autophosphorylation, which decreases in the presence of the substrate. *Lower panels* are loading controls of FIP3 (anti-GST) or aPKC (anti-aPKC). Blots were probed with the indicated antibodies. (JPG 441 kb) Additional file 2: Figure S2. Nuf subcellular distribution is affected in the absence of aPKC. a-c. Confocal images of wing discs containing *aPKC* clones marked by the absence of aPKC (green). (a) In the absence of aPKC, Crb (a’ and red in a), although affected, remains partially in the membrane of some cells and Nuf accumulates apically (a” and blue in a). (b-c) Loss of aPKC does not affect the subcellular distribution of the adherents junction marker DE-Cad (b’ and red in b) or Par3 (c’ and red in c) although Nuf accumulates (blue in b, c and b”, c”). d-e. Confocal images of wing discs of *baz* ^*4*^ *FRT19A::FRT19A_armLZ* larvae (d) and *Crb* ^*1*^ *FRT82B::FRT82B_UbiGFP* (e). In the absence of Baz or Crb (marked by the absence of Baz or Crb, green in d and e, respectively), aPKC can be detected in the membrane although at lower levels (red in d and e and grey d’ and e’) and the subcellular distribution of Nuf is not affected (blue in d and e and grey d” and c”). *Lower panels* are transversal views of the epithelia in the *upper panels* and double-head arrows mark the extension of the clone region. On the *lower panels* A marks apical and B basal. Scale bars 10 μm. (JPG 995 kb) Additional file 3: Figure S3. Nuf phosphorylation does not affect its partners-binding. a. *Left panel*, Rab11 binding assay. Wild-type Nuf and mutated S155A or S155D proteins were incubated with recombinant GST-Rab11 and pulled down with anti-GST beads. Immunoblotting of the complexes with anti-Nuf shows no variation in the interaction with Rab11. In the *right panel*, increasing amounts of aPKC were added to the GST-Rab11 :: Nuf incubation mixture. No competition was detected. b-c. Nuf binding to microtubule motor proteins. *Left panels*, recombinant GST-Nuf- wild-type and mutated S155A or S155D proteins were used in pull-down assays from embryonic extracts. Immunoblotting of the complexes and probing with anti-Kinesin (b) or anti-Dynactin (c) showed aPKC-phosphorylation-independent Nuf binding to Kinesin or Dynactin proteins. *Right panels*, competition assays with aPKC. Increasing amounts of aPKC were used in the binding assays. No variations in affinities were detected. d. Competition assay of aPKC with Dynein Light Chain, DLIC, for Nuf binding. The three variants of Nuf bind to DLIC and increasing amounts of aPKC cannot displace this binding. e. Schematic representation of Nuf binding to Dynein and Kinesin complex. f. Wild-type Nuf binds to 14-3-3 protein from embryonic extracts. NufS155A and S155D binding to 14-3-3 can also be detected at lower levels. g. Nuf binding to 14-3-3 is independent of aPKC phosphorylation. The binding affinity of Nuf to 14-3-3 did not change after in vitro phosphorylation of Nuf by aPKC. *Lower panels* are loading controls in A, B and C. 10 % of the beads used in each pull-down assay were run in a gel and probed with anti-GST. *Middle panel* in G shows the phosphorylation of Nuf by aPKC. (JPG 1090 kb) Additional file 4: Figure S4. Overexpression of S155 versions of Nuf does not affect Crb or Par-3 levels. a-b. Wing discs of *nuf* ^*1*^ */+* larvae overexpressing in the posterior cells (*hh-Gal4* driver) Myc-Nuf^S155A^ or Myc-Nuf^S155D^ and stained for Crb (a, green), Par-3 (b, green) or Nuf (a-b anti-Myc, magenta). Quantification of Crb (a) and Par-3 (b) levels across the cell membrane of posterior (green and blue lines) and anterior (orange and red lines) cells are shown in the graphs, expressed in arbitrary units. c. Quantification, in arbitrary units, of aPKC (*upper panels*) or DE-Cad (*lower panels*) levels in *nuf* ^*1*^ homozygotic larvae overexpressing in the posterior cells of imaginal wing discs (*en-Gal4* driver) Myc-Nuf^WT^ (*left*), Myc-Nuf^S155A^ *(middle*) or Myc-Nuf^S155D^ (right). Posterior cells are represented with blue (aPKC graphs) or green (DE-Cad graphs) lines and anterior cells with red (aPKC graphs) or orange (DE-Cad graphs) lines. Consistent with the quantification in heterozygous background, only aPKC levels increase when overexpressing Nuf^S155A^. d. The membrane levels obtained in the quantification of aPKC (Fig. 3c and Additional file 3: Figure S3c) or DECad (Fig. 3e and Additional file 3: Figure S3c) in anterior and posterior cells were plotted. aPKC levels show statistically significant increases in both heterozygous and homozygous *nuf* mutant backgrounds. (JPG 1693 kb) Additional file 5: Figure S5. An amino terminal truncated form of nuf (nuf^BRW^) colocalizes with aPKC and Rab11. Imaginal wing discs overexpressing a truncated form of Nuf (BRW) lacking the N terminal region (1–213 aa) drive by *hh-G4* recombined with *nuf* ^*1*^. This generates a genetic background where there is no wild-type Nuf protein (*nuf* ^*1*^ */nuf* ^*BRW*^). Vesicles colocalizing aPKC (green), Nuf (red) and Rab11 (blue) can be detected (*arrows*). Three sections (projections of five layers each) at different levels, from an apical to a more basal position, are shown. Sagittal views of each are *below*, *yellow arrows* mark the position where sagittal sections were taken (*asterisks*). Scale bar 10 μm. (JPG 1143 kb) Additional file 6: Figure S6. Clones of nuf don’t affect DE-Cad, Par3 of PATJ apical determinants. a. DE-Cad (red) staining in *nuf* ^*KG00314*^ (*upper panels*) or *nuf* ^*1*^ (*lower panels*) mutant clones. Graphics at the right show fluorescence levels of DE-Cad comparing wild-type (red) with clone cells (blue) for *nuf* ^*KG00314*^ (*upper*) or *nuf* ^*1*^ (*lower*). b. Staining of Par3 (red) in *nuf* ^*KG00314*^ (*upper panels*) or *nuf* ^*1*^ (*lower panels*) mutant clones. Graphics at the right show fluorescence levels of Par3 comparing wild-type (red) with clone cells (blue) for *nuf* ^*KG00314*^ (*upper*) or *nuf* ^*1*^ (*lower*). c. Staining of the apical marker DPATJ (red) in *nuf* ^*KG00314*^ (*upper panels*) or *nuf* ^*1*^ (*lower panels*) mutant clones. Graphics at the right show fluorescence levels of DPATJ comparing wild-type (red) with clone cells (blue) for *nuf* ^*KG00314*^ (*upper*) or *nuf* ^*1*^ (*lower*). Clones are marked by the absence of β-gal in green. *Lower panels* show sagittal views of the clones. Scale bars 10 μm. (JPG 1184 kb) Additional file 7: Supplementary methods. (DOC 68 kb)

## Abbreviations

aPKC: Atypical protein kinase C
ARE: Apical recycling endosomes
Crb: Crumbs
DPATJ: Drosophila Pals1 associated tight junctions
E-Cad: E-cadherin
FIPs: Family interacting proteins
KD: Kinase domain
Nuf: Nuclear fallout
RD: Regulatory domain
RE: Recycling endosomes
REdv: Recycling endosomes derived vesicles
