# Supplementary figures and images for: Nuclear fallout provides a new link between aPKC and polarized cell trafficking
Source: BMC Biol. 2016 Apr 18;14:32. doi: 10.1186/s12915-016-0253-6 (PMC4836198; doi:10.1186/s12915-016-0253-6)

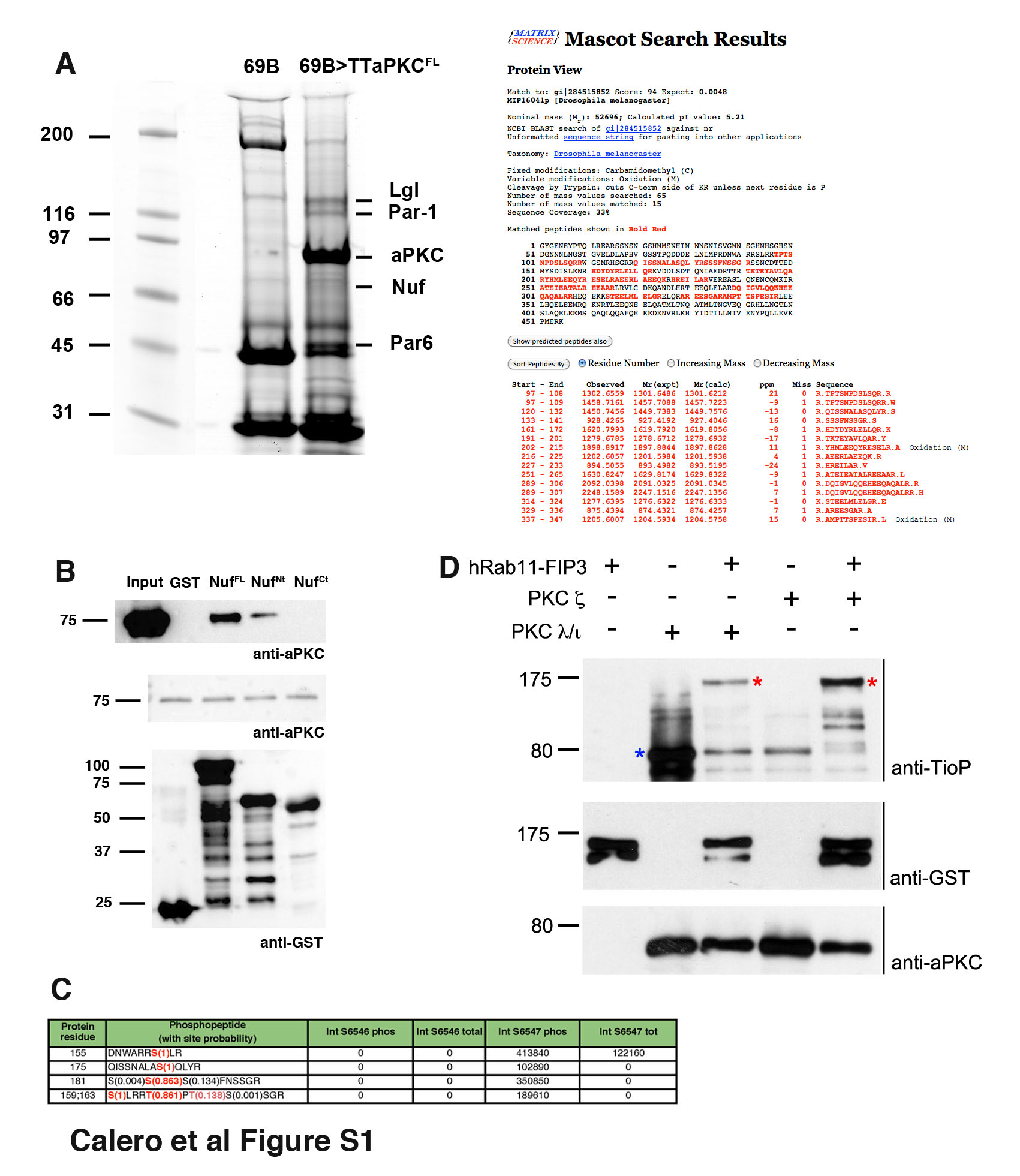

Supplement: Additional file 1: Figure S1. — Tandem Affinity Purification of aPKC. aPKC-Nuf interaction and phosphorylation. a. EZ-Blue staining of a gel loaded with the elution fractions obtained after Tandem Affinity Purification of aPKC from control embryonic extract (69B-Gal4) or embryos overexpressing TTaPKC FL (69B-Gal4 driver). Nuf and known aPKC interacting proteins (Par-6, Par-1 and Lgl) were identified. Peptides corresponding to Nuf identified by mass spectrometry analysis are shown on the right. b. Pull-down assays from Drosophila embryonic protein extracts with GST, GST-Nuf FL or GST fused to the amino (Nt) or carboxy (Ct) terminal domains of Nuf. Immunoblotting of bound proteins with anti-aPKC shows the interaction of Nuf full-length and the amino terminal but not the carboxy terminal region of Nuf with aPKC. The first lane (Input) contains 10 % of the extract used in the assay. In the middle panel, extract was probed with anti-α-aPKC as a loading control. Lower panel: 10 % of the beads used in each pull-down assay were run in a gel and probed with anti-GST. c. To gain more insight into the phosphopeptide-to-protein mapping, we used two widely used software programs for peptide identification, namely MSGF+ and MaxQuant. While the former provides better protein sequence coverage, MaxQuant is more statistically robust. Note that phosphorylation of S155 on NUF was detected on both phosphopeptide-enriched and non-enriched samples while the other four phosphorylation sites of Nuf (S175, S181, S159, T163) were detected only after enrichment. Given the fact that LC-MS/MS system provides low attomoles sensitivity, it is likely that the stoichiometry of these phosphopeptides is extremely low. d. Rab11-FIP3, human orthologous of Nuf, is phosphorylated by human aPKCs, aPKC ζ and λ/ι, red asterisks. Blue asterisk marks aPKC autophosphorylation, which decreases in the presence of the substrate. Lower panels are loading controls of FIP3 (anti-GST) or aPKC (anti-aPKC). Blots were probed with the indicated antibo [file 12915_2016_253_MOESM1_ESM.jpg]

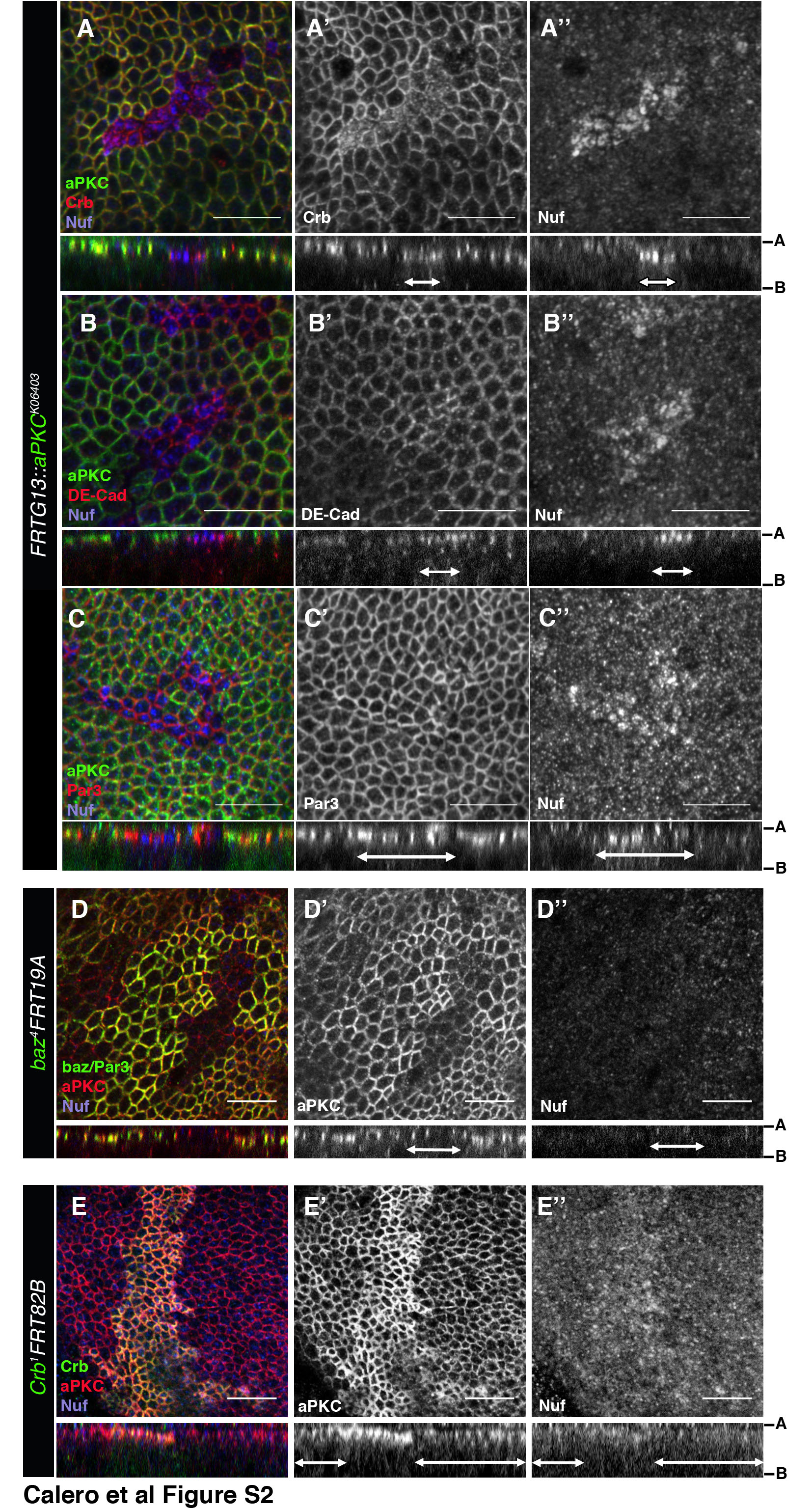

Supplement: Additional file 2: Figure S2. — Nuf subcellular distribution is affected in the absence of aPKC. a-c. Confocal images of wing discs containing aPKC clones marked by the absence of aPKC (green). (a) In the absence of aPKC, Crb (a’ and red in a), although affected, remains partially in the membrane of some cells and Nuf accumulates apically (a” and blue in a). (b-c) Loss of aPKC does not affect the subcellular distribution of the adherents junction marker DE-Cad (b’ and red in b) or Par3 (c’ and red in c) although Nuf accumulates (blue in b, c and b”, c”). d-e. Confocal images of wing discs of baz 4 FRT19A::FRT19A_armLZ larvae (d) and Crb 1 FRT82B::FRT82B_UbiGFP (e). In the absence of Baz or Crb (marked by the absence of Baz or Crb, green in d and e, respectively), aPKC can be detected in the membrane although at lower levels (red in d and e and grey d’ and e’) and the subcellular distribution of Nuf is not affected (blue in d and e and grey d” and c”). Lower panels are transversal views of the epithelia in the upper panels and double-head arrows mark the extension of the clone region. On the lower panels A marks apical and B basal. Scale bars 10 μm. (JPG 995 kb) [file 12915_2016_253_MOESM2_ESM.jpg]

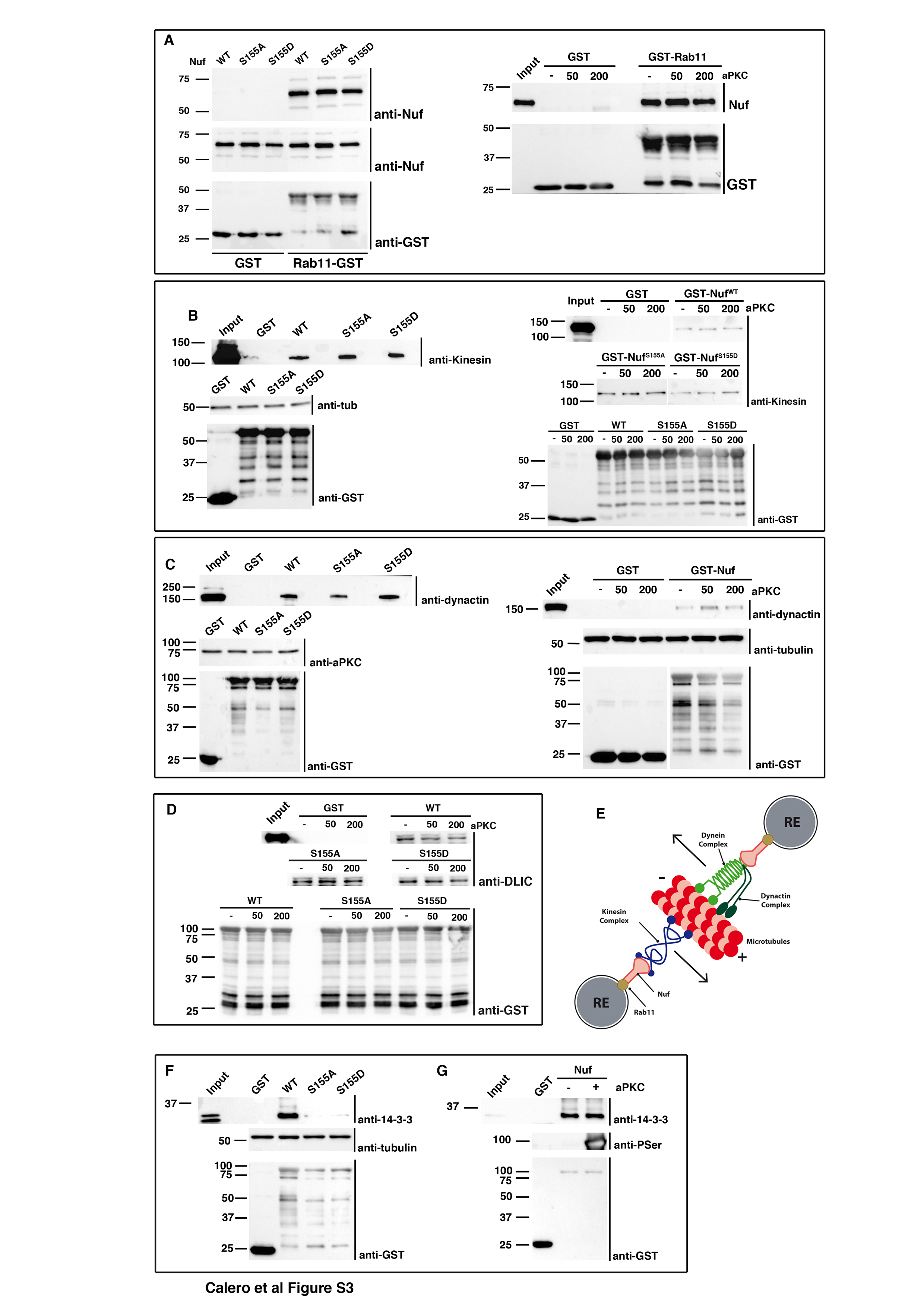

Supplement: Additional file 3: Figure S3. — Nuf phosphorylation does not affect its partners-binding. a. Left panel, Rab11 binding assay. Wild-type Nuf and mutated S155A or S155D proteins were incubated with recombinant GST-Rab11 and pulled down with anti-GST beads. Immunoblotting of the complexes with anti-Nuf shows no variation in the interaction with Rab11. In the right panel, increasing amounts of aPKC were added to the GST-Rab11 :: Nuf incubation mixture. No competition was detected. b-c. Nuf binding to microtubule motor proteins. Left panels, recombinant GST-Nuf- wild-type and mutated S155A or S155D proteins were used in pull-down assays from embryonic extracts. Immunoblotting of the complexes and probing with anti-Kinesin (b) or anti-Dynactin (c) showed aPKC-phosphorylation-independent Nuf binding to Kinesin or Dynactin proteins. Right panels, competition assays with aPKC. Increasing amounts of aPKC were used in the binding assays. No variations in affinities were detected. d. Competition assay of aPKC with Dynein Light Chain, DLIC, for Nuf binding. The three variants of Nuf bind to DLIC and increasing amounts of aPKC cannot displace this binding. e. Schematic representation of Nuf binding to Dynein and Kinesin complex. f. Wild-type Nuf binds to 14-3-3 protein from embryonic extracts. NufS155A and S155D binding to 14-3-3 can also be detected at lower levels. g. Nuf binding to 14-3-3 is independent of aPKC phosphorylation. The binding affinity of Nuf to 14-3-3 did not change after in vitro phosphorylation of Nuf by aPKC. Lower panels are loading controls in A, B and C. 10 % of the beads used in each pull-down assay were run in a gel and probed with anti-GST. Middle panel in G shows the phosphorylation of Nuf by aPKC. (JPG 1090 kb) [file 12915_2016_253_MOESM3_ESM.jpg]

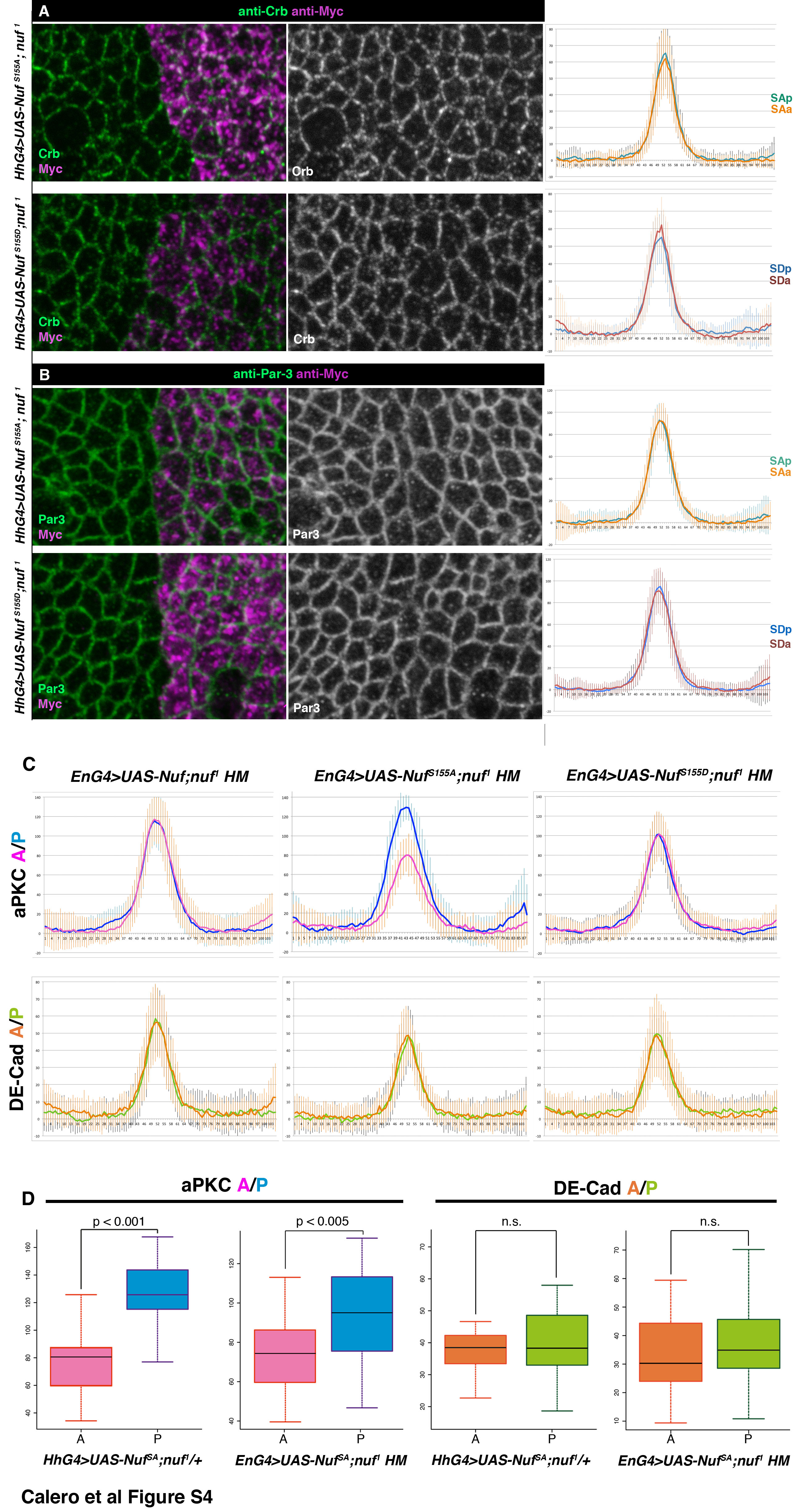

Supplement: Additional file 4: Figure S4. — Overexpression of S155 versions of Nuf does not affect Crb or Par-3 levels. a-b. Wing discs of nuf 1 /+ larvae overexpressing in the posterior cells (hh-Gal4 driver) Myc-NufS155A or Myc-NufS155D and stained for Crb (a, green), Par-3 (b, green) or Nuf (a-b anti-Myc, magenta). Quantification of Crb (a) and Par-3 (b) levels across the cell membrane of posterior (green and blue lines) and anterior (orange and red lines) cells are shown in the graphs, expressed in arbitrary units. c. Quantification, in arbitrary units, of aPKC (upper panels) or DE-Cad (lower panels) levels in nuf 1 homozygotic larvae overexpressing in the posterior cells of imaginal wing discs (en-Gal4 driver) Myc-NufWT (left), Myc-NufS155A (middle) or Myc-NufS155D (right). Posterior cells are represented with blue (aPKC graphs) or green (DE-Cad graphs) lines and anterior cells with red (aPKC graphs) or orange (DE-Cad graphs) lines. Consistent with the quantification in heterozygous background, only aPKC levels increase when overexpressing NufS155A. d. The membrane levels obtained in the quantification of aPKC (Fig. 3c and Additional file 3: Figure S3c) or DECad (Fig. 3e and Additional file 3: Figure S3c) in anterior and posterior cells were plotted. aPKC levels show statistically significant increases in both heterozygous and homozygous nuf mutant backgrounds. (JPG 1693 kb) [file 12915_2016_253_MOESM4_ESM.jpg]

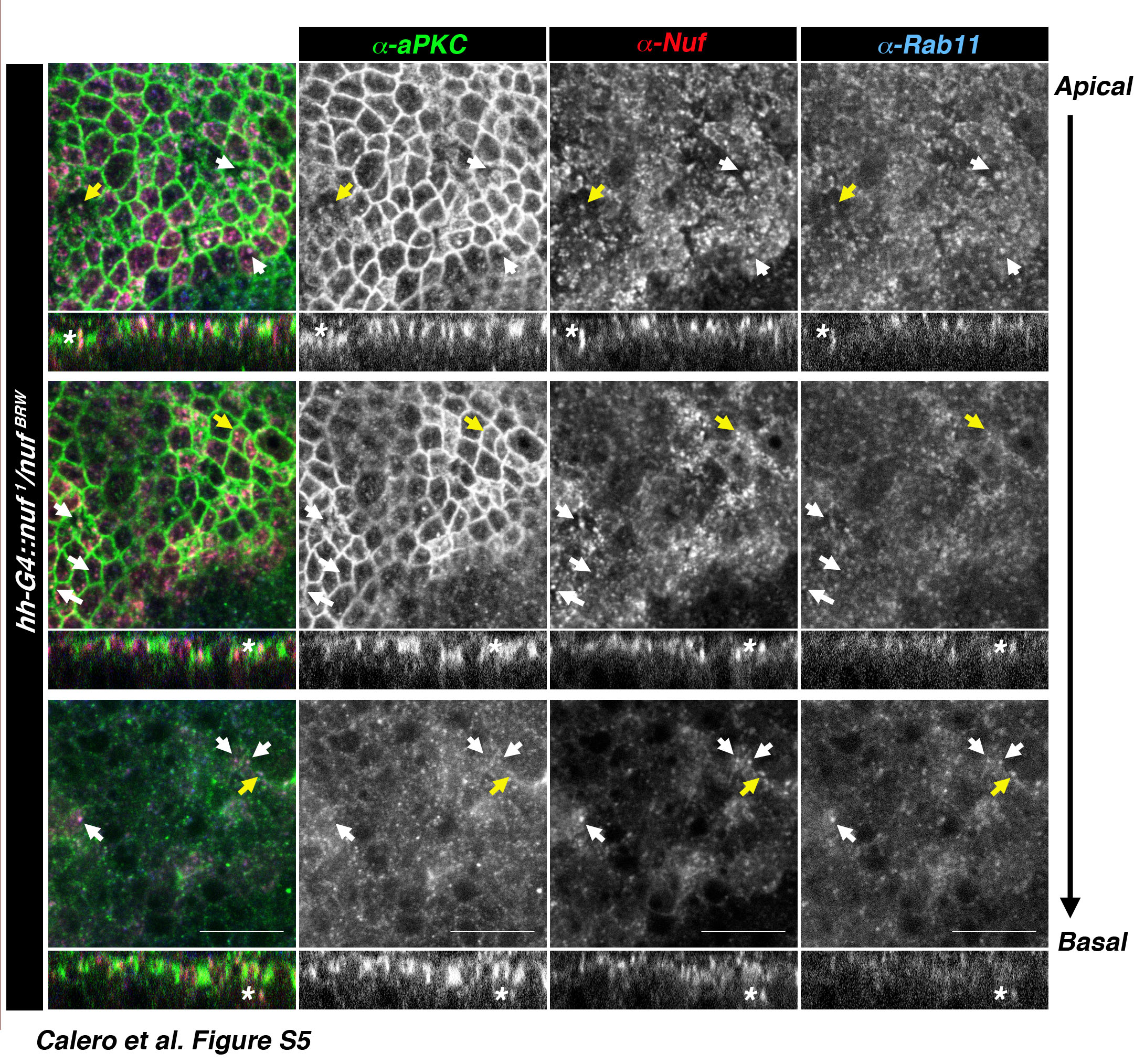

Supplement: Additional file 5: Figure S5. — An amino terminal truncated form of nuf (nufBRW) colocalizes with aPKC and Rab11. Imaginal wing discs overexpressing a truncated form of Nuf (BRW) lacking the N terminal region (1–213 aa) drive by hh-G4 recombined with nuf 1. This generates a genetic background where there is no wild-type Nuf protein (nuf 1 /nuf BRW). Vesicles colocalizing aPKC (green), Nuf (red) and Rab11 (blue) can be detected (arrows). Three sections (projections of five layers each) at different levels, from an apical to a more basal position, are shown. Sagittal views of each are below, yellow arrows mark the position where sagittal sections were taken (asterisks). Scale bar 10 μm. (JPG 1143 kb) [file 12915_2016_253_MOESM5_ESM.jpg]

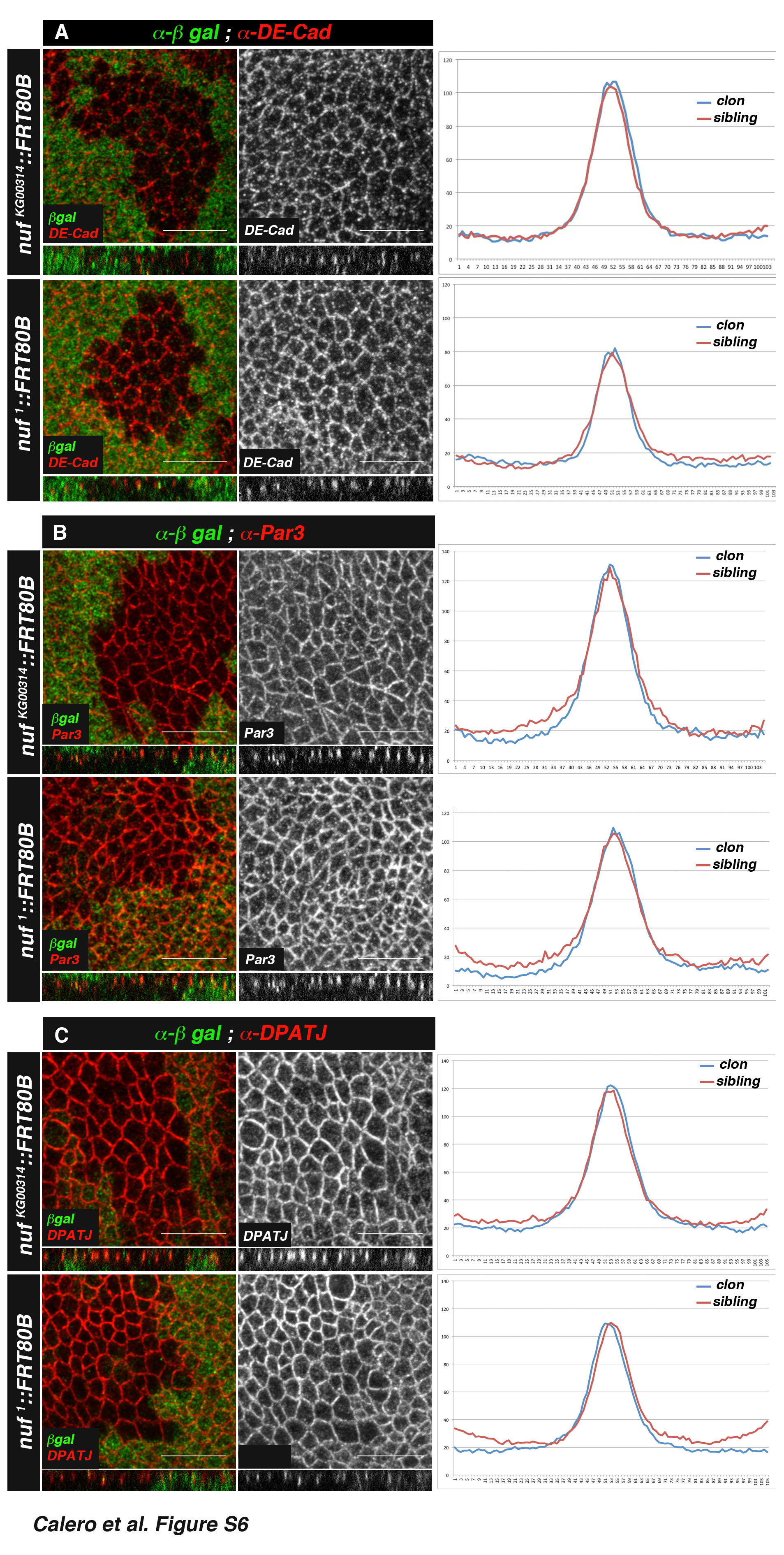

Supplement: Additional file 6: Figure S6. — Clones of nuf don’t affect DE-Cad, Par3 of PATJ apical determinants. a. DE-Cad (red) staining in nuf KG00314 (upper panels) or nuf 1 (lower panels) mutant clones. Graphics at the right show fluorescence levels of DE-Cad comparing wild-type (red) with clone cells (blue) for nuf KG00314 (upper) or nuf 1 (lower). b. Staining of Par3 (red) in nuf KG00314 (upper panels) or nuf 1 (lower panels) mutant clones. Graphics at the right show fluorescence levels of Par3 comparing wild-type (red) with clone cells (blue) for nuf KG00314 (upper) or nuf 1 (lower). c. Staining of the apical marker DPATJ (red) in nuf KG00314 (upper panels) or nuf 1 (lower panels) mutant clones. Graphics at the right show fluorescence levels of DPATJ comparing wild-type (red) with clone cells (blue) for nuf KG00314 (upper) or nuf 1 (lower). Clones are marked by the absence of β-gal in green. Lower panels show sagittal views of the clones. Scale bars 10 μm. (JPG 1184 kb) [file 12915_2016_253_MOESM6_ESM.jpg]
