# Supplementary material for: Nuclear fallout provides a new link between aPKC and polarized cell trafficking
Source: BMC Biol. 2016 Apr 18;14:32. doi: 10.1186/s12915-016-0253-6 (PMC4836198; doi:10.1186/s12915-016-0253-6)
Supplement: Additional file 7: — Supplementary methods. (DOC 68 kb) [file 12915_2016_253_MOESM7_ESM.doc]

**Appendix Figure Legends.**

*Additional File1: Figure S1.* ***Tandem Affinity Purification of aPKC. aPKC-Nuf interaction and phosphorylation***.

**a.** EZ-Blue staining of a gel loaded with the elution fractions obtained after Tandem Affinity Purification of aPKC from control embryonic extract (69B-Gal4) or embryos overexpressing TTaPKC FL (*69B-Gal4* driver). Nuf and known aPKC interacting proteins (Par-6, Par-1 and Lgl) were identified along. Peptides corresponding to Nuf identified by mass spectrometry analysis are shown on the right.

**b.** Pull-down assays from *Drosophila* embryonic protein extracts with GST, GST-Nuf FL or GST fused to the amino (Nt) or carboxy (Ct) terminal domains of Nuf. Immunoblotting of bound proteins with anti-aPKC shows the interaction of Nuf full-length and the amino terminal but not the carboxy terminal region of Nuf with aPKC. First lane (Input) contains 10% of the extract used in the assay. In the middle panel, extract was probed with anti-α-aPKC as a loading control. Lower panel: 10% of the beads used in each pull-down assay were run in a gel and probed with anti-GST.

**c.** To gain more insight into the phosphopeptide-to-protein mapping, we used 2 widely used software for peptide identification, namely MSGF+ and MaxQuant. While the former provides better protein sequence coverage, MaxQuant is more statistically robust. Note that phosphorylation of S155 on NUF was detected on both phosphopeptide-enriched and non-enriched samples. While the other 4 phosphorylation sites of Nuf (S175, S181, S159, T163) were detected only after enrichment. Given the fact that LC-MS/MS system provides low attomoles sensitivity, it is likely that the stoichiometry of these phosphopeptides is extremely.

**d.** Rab11-FIP3, human orthologous of Nuf, is phosphorylated by human aPKCs, aPKC ζ and λ/ι, red asterisks. Blue asterisk marks aPKC autophosphorylation, which decreases in the presence of the substrate. Lower panels are loading controls of FIP3 (anti-GST) or aPKC (anti-aPKC). Blots were probed with the indicated antibodies.

*Additional File 2: Figure S2.* ***Nuf subcellular distribution is affected in the absence of* *aPKC*.**

**a-c.** Confocal images of wing discs containing *aPKC* clones marked by the absence of aPKC (green). (a) In the absence of aPKC, Crb (a’ and red in a) although affected remains partially in the membrane of some cells and Nuf accumulates apically (a’’ and blue in a). (b-c) Loss of aPKC does not affect the subcellular distribution of the adherents junction marker DE-Cad (b’ and red in b) or Par3 (c’ and red in c) although Nuf accumulates (blue in b, c and b’’, c’’).

**d-e.** Confocal images of wing discs of *baz^4^FRT19A::FRT19A_armLZ* larvae (d) and *Crb^1^FRT82B::FRT82B_UbiGFP* (e). In the absence of Baz or Crb (marked by the absence of Baz or Crb, green in d and e respectively), aPKC can be detected in the membrane although at lower levels (red in d and e and grey d’ and e’) and the subcellular distribution of Nuf is not affected (blue in d and e and grey d’’ and c’’). Lower panels are transversal views of the epithelia in the upper panels and double-head arrows mark the extension of the clone region. On the lower panels A marks apical and B basal. Scale bars 10 μm.

*Additional File 3: Figure S3.* ***Nuf phosphorylation does not affect its partners-binding***.

**a.** Left panel, Rab11 binding assay. Wild-type Nuf and mutated S155A or S155D proteins were incubated with recombinant GST-Rab11 and pulled down with anti-GST beads. Immunoblotting of the complexes with anti-Nuf shows no variation in the interaction with Rab11. In the right panel, increasing amounts of aPKC were added to the GST-Rab11 :: Nuf incubation mixture. No competition was detected.

**b-c.** Nuf binding to microtubule motor proteins. Left panels, recombinant GST-Nuf- wild-type and mutated S155A or S155D proteins were used in pull-down assays from embryonic extracts. Immunoblotting of the complexes and probing with anti-Kinesin (b) or anti-Dynactin (c) showed aPKC-phosphorylation-independent Nuf binding to Kinesin or Dynactin proteins. Right panels, competition assays with aPKC. Increasing amounts of aPKC were used in the binding assays. No variations in affinities were detected.

**d.** Competition assay of aPKC with Dynein Light Chain, DLIC, for Nuf binding. The three variants of Nuf bind to DLIC and increasing amounts of aPKC cannot displace this binding.

**e.** Schematic representation of Nuf binding to Dynein and Kinesin complex.

**f.** Wild-type Nuf binds to 14-3-3 protein from embryonic extracts. NufS155A and S155D binding to 14-3-3 can also be detected at lower levels.

**g.** Nuf binding to 14-3-3 is independent of aPKC phosphorylation. The binding affinity of Nuf to 14-3-3 did not change after *in vitro* phosphorylation of Nuf by aPKC.

Lower panels are loading controls in A, B and C. 10% of the beads used in each pull-down assay were run in a gel and probed with anti-GST. Middle panel in G shows the phosphorylation of Nuf by aPKC.

*Additional File 4: Figure S4.* ***Overexpression of*** ***S155 versions of Nuf does not affect Crb or Par-3 levels***.

**a-b.** Wing discs of *nuf^1^/+* larvae overexpressing in the posterior cells (*hh-Gal4* driver) Myc-Nuf^S155A^ or Myc-Nuf^S155D^ and stained for Crb (a, green), Par-3 (b, green) or Nuf (a-b anti-Myc, magenta). Quantification of Crb (a) and Par-3 (b) levels across the cell membrane of posterior (green and blue lines) and anterior (orange and red lines) cells are shown in the graphs, expressed in arbitrary units.

**c.** Quantification, in arbitrary units, of aPKC (upper panels) or DE-Cad (lower panels) levels in *nuf^1^* homozygotic larvae overexpressing in the posterior cells of imaginal wing discs (*en-Gal4* driver) Myc-Nuf^WT^ (left), Myc-Nuf^S155A^ (middle) or Myc-Nuf^S155D^ (right). Posterior cells were represented with blue (aPKC graphs) or green (DE-Cad graphs) lines and anterior cells with red (aPKC graphs) or orange (DE-Cad graphs) lines. Consistently with the quantification in heterozygous background, only aPKC levels increases when overexpressing Nuf^S155A^.

**d.** The membrane levels obtained in the quantification of aPKC (Fig. 3c and S3c) or DECad (Fig. 3e and S3c) in anterior and posterior cells were plotted. aPKC levels show a statistically significant increases in both heterozygous and homozygous *nuf* mutant backgrounds.

*Additional File 5: Figure S5.* ***An amino terminal truncated form of nuf (nuf^BRW^) colocalizes with aPKC and Rab11.***

Imaginal wing discs overexpressing a truncated form of Nuf (BRW) lacking the N terminal region (1-213 aa) drive by *hh-G4* recombined with *nuf^1^*. This generates a genetic background where there is no wild-type Nuf protein (*nuf^1^/nuf^BRW^*). Vesicles colocalizing aPKC (green), Nuf (red) and Rab11 (blue) can be detected (arrows). Three sections (projections of 5 layers each) at different levels, from an apical to a more basal position, are shown. Sagittal views of each are below, yellow arrows mark the position where sagittal sections were taken (asterisks). Scale bar 10 μm

*Additional File 6: Figure S6.* ***Clones of nuf don’t affect DE-Cad, Par3 of PATJ apical determinants.***

**a.** DE-Cad (red) staining in *nuf^KG00314^* (upper panels) or *nuf^1^* (lower panels) mutant clones. Graphics at the right show fluorescence levels of DE-Cad comparing wild-type (red) with clone cells (blue) for *nuf^KG00314^* (upper) or *nuf^1^* (lower).

**b.** Staining of Par3 (red) in *nuf^KG00314^* (upper panels) or *nuf^1^* (lower panels) mutant clones. Graphics at the right show fluorescence levels of Par3 comparing wild-type (red) with clone cells (blue) for *nuf^KG00314^* (upper) or *nuf^1^* (lower).

**c.** Staining of the apical marker DPATJ (red) in *nuf^KG00314^* (upper panels) or *nuf^1^* (lower panels) mutant clones. Graphics at the right show fluorescence levels of DPATJ comparing wild-type (red) with clone cells (blue) for *nuf^KG00314^* (upper) or *nuf^1^* (lower).

Clones are marked by the absence of β-gal in green. Lower panels show sagittal views of the clones. Scale bars 10 μm

**Additional Methods.**

**Fly strains.** We used the following stocks: Oregon R (wild-type), *nuf^1^* [1], *nuf^KG00314^* (flybase), *nuf^BRW^* [2], *FRTG13::aPKC^K06403^*, *baz^4^::FRT19a*, *FRT82B::crb^1^*, *sec6^∆20^::FRTG13, /+* [3], *sec5^E^::FRT40A* [4]. UAS-Rab11-RNAi (v22198) from the Vienna *Drosophila* RNAi Center.

We used 69B*-Gal4*, *24B-Gal4*, *en-Gal4* and *Hh-Gal4* as driver lines.

Clones in imaginal disc were generated at 48-72 and 72-96 h after egg laying in the following genotypes:

- *hsFLP/+; FRTG13::aPKC^k06403^/ FRTG13::Ubi-GFP*;
- *baz^4^::FRT19A/ FRT19A::armLacZ; HSFlip/+*;
- *HSFlip/+; FRT82B::crb^1^/;FRT82B::Ubi.GFP;*
- *hsFLP/+; sec5^E^::FRT40A/ FRT40A::armLZ;*
- *hsFLP/+; sec6^∆20^::FRTG13/ FRTG13::Ubi-GFP;*
- *hsFLP/+; nuf^1^::FRT80B/armLZ ::FRT80B;*
- *hsFLP/+; nuf^KG00314^::FRT80B/armLZ ::FRT80B;*
- *hsFLP/+; actin>y>G4::UAS-GFP/UAS-Rab11-RNAi;*
- *hsFLP/+; FRTG13::aPKC^k06403^/ FRTG13::Ubi-GFP*; *HhG4/UAS-Myc-Nuf^S155A^.*

The imaginal wing discs staining were performed from the following crosses:

- *Hh-G4/TM6B Hu X UAS-Myc-Nuf^WT^; nuf^1^/TM6B Hu* => *HhG4/UAS-Myc-Nuf^WT^; nuf^1^/+* larvae were selected by the absence of Hu dominant marker.
- *Hh-G4/TM6B Hu X UAS-Myc-Nuf^S155A^; nuf^1^/TM6B Hu* => *HhG4/UAS-Myc-Nuf^S155A^; nuf^1^/+* larvae were selected by the absence of Hu dominant marker.
- *Hh-G4/TM6B Hu X UAS-Myc-Nuf^S155D^; nuf^1^/TM6B Hu* => *HhG4/UAS-Myc-Nuf^S155D^; nuf^1^/+* larvae were selected by the absence of TM6B marker.
- *enG4/Cyo; nuf^1^/TM6B Hu X UAS-Myc-Nuf^WT^; nuf^1^/TM6B Hu* => *enG4/ UAS-Myc-Nuf^WT^; nuf^1^/nuf^1^* larvae were selected by the absence of Hu dominant marker.
- *enG4/Cyo; nuf^1^/TM6B Hu X UAS-Myc-Nuf ^S155A^; nuf^1^/TM6B Hu* => *enG4/ UAS-Myc-Nuf^S155A^; nuf^1^/nuf^1^* larvae were selected by the absence of Hu dominant marker.
- *enG4/Cyo; nuf^1^/TM6B Hu X UAS-Myc-Nuf ^S155D^; nuf^1^/TM6B Hu* => *enG4/ UAS-Myc-Nuf^S155D^; nuf^1^/nuf^1^* larvae were selected by the absence of Hu dominant marker.
- *Hh-G4::nuf^1^/TM6b Hu X nuf^BRW^/TM6B Hu* => *HhG4::nuf^1^/ nuf^BRW^* larvae were selected by the absence of Hu dominant marker.

**Expression plasmids.**

The following constructs were used:

pGEX-6P-1-Nuf FL [5].

pGEX-2T-Nuf-NH and pGEX-2T-Nuf-CO: nuf cDNA fragments encoding the amino-terminal part of Nuf (Nuf-NH) or the carboxy-terminal domain (Nuf-CO) were amplified from *nuf* cDNA clone RE46851 using the primers NufNH forward, for (BamHI) and NufNH reverse, rev (BamHI) and NufCO for (BamHI) and NufCO rev (BamHI), respectively. After sequencing, the cDNAs fragments were subcloned into the BamHI site of pGEX-2T (GE-Healthcare).

pGEX-2T-Nuf-NH mutants: Mutagenesis of Nuf-NH was performed by the site directed Quick-Change system (Stratagene) using pGEMt-easy-NufNH WT as template using the primer pairs: Nuf S155A for and Nuf S155A rev; Nuf S155D for and Nuf S155D rev; Nuf S180-2A for and Nuf S180-2A rev; Nuf S175A for and Nuf S175A rev; Nuf T159A for and Nuf T159A rev. Mutagenesis was confirmed by sequencing. Fragments were subcloned in the BamHI site of pGEX-2T

pGEX-2T-Nuf-FL mutants: Fragments (BamHI/BamHI) of pUASp-Nuf constructs containing mutation S155A or S155D were used for substitution of the BamHI/BamHI fragment into the pGEX-2T.

pGEX-6P-1-Nuf-FL-mutants: Fragments (BamHI/HindIII) containing mutation S155A or S155D from pGEX-2T-Nuf were used for substitution of the BamHI/HindIII fragment into the pGEX-6P-1-Nuf FL WT.

pGEX-2T-Rab11: Fragment was amplified from *Rab11* cDNA clone RE11886 using primers Rab11Bam5’ and Rab11Bam3’ and after sequencing, the cDNAs were subcloned into the BamHI sites of pGEX-2T.

pGEX-3X-Rab11-FIP3 [6].

pGEX-6P-1-DLIC [5].

pGEX-6P-1-14-3-3ε: 14-3-3ε ORF was cloned from pMAL-c::14-3-3ε [7] into pGEX-6P-1 plasmid in BamHI/SalI target sites.

**UAS-constructs.**

*pUASp-6xmyc-Nuf* constructs: Fragments (NotI/HindIII) from pGEMt-easy-NufNH WT or mutated forms were cloned into NotI/HindIII of pFLC-I-*nuf* (clone RE46851) for obtaining WT and mutants versions of Nuf without 5’UTR and a BamHI target site before the first ATG.

Fragments (BamHI/BamHI) from pFLC-I-Nuf-FL-WT, pFLC-I-Nuf-FL-mut S155A or pFLC-I-Nuf-FL-mut S155D were cloned into the pUASp vector. 6xMyc tag was included in 5’ at the SpeI restriction site.

pUASt-TT constructs: EcoRI/NotI fragment from pCASPER-TT [8] containing the TetraTag (TT) cassette was cloned in pUASt into the EcoRI/NotI sites.

Fragments from aPKC-FL, aPKC-RD and aPKC-KD were amplified using *aPKC* cDNA (AY118402 clone) as template and combinations of NotI-aPKC FL5’, XbaI-aPKC FL3’, NotI-aPKC KD5’ and XbaI-aPKC RD3’ primers, cloned into pGEMt-easy and sequenced. The fragments (NotI-NotI) were cloned at the NotI site of pUASt-TT maintaining the reading frame with the TT.

The Kinase Dead (K293W) mutation of aPKC was generated by site-directed mutagenesis using the primer pairs *aPKC* mut for and *aPKC* rev and the pGEMt-easy aPKC FL and KD constructs as templates, confirmed by sequencing and subcloned in pUASt-TT as NotI/NotI (aPKC FL-mut) and NotI/XbaI (aPKC KD-mut).

Table of primers

| NufNH for (BamHI) | TTGGATCCATGGCACCAATGCCC |
| --- | --- |
| NufNH rev (BamHI) | TTGGATCCTTCGGTCTTGGTGCG |
| NufCO for (BamHI) | TTGGATCCTATGCAGTCCTTCAGGCG |
| NufCO rev (BamHI) | TTGGATCCCTACTTTCGCTCCATGGG |
| Rab11Bam5' | AAGGATCCATGGGTGCAAGAGAAGACG |
| Rab11Bam3' | AAGGATCCTCACTGACAGCACTGTTTGCGC |
| Nuf S155A for | GGGCCAGGCGCGCTCTGCGACGAACTCC |
| Nuf S155A rev | GGAGTTCGTCGCAGAGCGCGCCTGGCCC |
| Nuf S155D for | GGGCCAGGCGCGATCTCCGACGAACTCC |
| Nuf S155D rev | GGAGTTCGTCGCAGATCGCGCCTGGCCC |
| Nuf S180-2A for | GCCAACTCTATAGAGCAGCGGCCTTTAACTCATCGGG |
| Nuf S180-2A rev | CCCGATGAGTTAAAGGCCGCTGCTCTATAGAGTTGGC |
| Nuf S175A for | GCCTTAGCCGCCCAACTCTATAGATCATCGAGC |
| Nuf S175A rev | GCTCGATGATCTATAGAGTTGGGCGGCTAAGGC |
| Nuf T159A for | CTGCGACGAGCTCCCACCAGTTCCG |
| Nuf T159A rev | AACTGGTGGGAGCTCGTCGCAGACTGC |
| XbaI-aPKC RD3’ | CGTCTAGATCAGTCGGTGCAGTGC |
| NotI-aPKC KD5’ | TAGCGGCCGCATGAGCGGCGGAGC |
| NotI-aPKC FL5’ | TAGCGGCCGCATGCAGAAAATGCCC |
| XbaI-aPKC FL3’ | CGTCTAGATCAGACGCAATCCTCC |
| aPKC mut for | CGCATCTACGCCATGTGGGTGATCAAGAAGG |
| aPKC mut rev | CCTTCTTGATCACCCACATGGCGTAGATGCG |

**Additional File 7 Refernces**

1. Rothwell WF, Fogarty P, Field CM, Sullivan W: Nuclear-fallout, a Drosophila protein that cycles from the cytoplasm to the centrosomes, regulates cortical microfilament organization. *Development* 1998, 125(7):1295-1303.

2. Singari S, Javeed N, Tardi NJ, Marada S, Carlson JC, Kirk S, Thorn JM, Edwards KA: Inducible protein traps with dominant phenotypes for functional analysis of the Drosophila genome. *Genetics* 2014, 196(1):91-105.

3. Beronja S, Laprise P, Papoulas O, Pellikka M, Sisson J, Tepass U: Essential function of Drosophila Sec6 in apical exocytosis of epithelial photoreceptor cells. *J Cell Biol* 2005, 169(4):635-646.

4. Langevin J, Morgan MJ, Sibarita JB, Aresta S, Murthy M, Schwarz T, Camonis J, Bellaiche Y: Drosophila exocyst components Sec5, Sec6, and Sec15 regulate DE-Cadherin trafficking from recycling endosomes to the plasma membrane. *Dev Cell* 2005, 9(3):365-376.

5. Otani T, Oshima K, Onishi S, Takeda M, Shinmyozu K, Yonemura S, Hayashi S: IKKepsilon regulates cell elongation through recycling endosome shuttling. *Dev Cell* 2011, 20(2):219-232.

6. Fielding AB, Schonteich E, Matheson J, Wilson G, Yu X, Hickson GR, Srivastava S, Baldwin SA, Prekeris R, Gould GW: Rab11-FIP3 and FIP4 interact with Arf6 and the exocyst to control membrane traffic in cytokinesis. *The EMBO journal* 2005, 24(19):3389-3399.

7. Benton R, St Johnston D: Drosophila PAR-1 and 14-3-3 inhibit Bazooka/PAR-3 to establish complementary cortical domains in polarized cells. *Cell* 2003, 115(6):691-704.

8. Yang P, Sampson HM, Krause HM: A modified tandem affinity purification strategy identifies cofactors of the Drosophila nuclear receptor dHNF4. *Proteomics* 2006, 6(3):927-935.
